# Chitosan Nanoparticles for Intranasal Drug Delivery

**DOI:** 10.3390/pharmaceutics16060746

**Published:** 2024-05-31

**Authors:** Hossein Omidian, Erma J. Gill, Sumana Dey Chowdhury, Luigi X. Cubeddu

**Affiliations:** Barry and Judy Silverman College of Pharmacy, Nova Southeastern University, Fort Lauderdale, FL 33328, USA; eg1262@mynsu.nova.edu (E.J.G.); sd2236@mynsu.nova.edu (S.D.C.); lcubeddu@nova.edu (L.X.C.)

**Keywords:** chitosan nanoparticles, intranasal drug delivery, neurodegenerative disorders, clinical translation, regulatory challenges

## Abstract

This manuscript explores the use of nanostructured chitosan for intranasal drug delivery, targeting improved therapeutic outcomes in neurodegenerative diseases, psychiatric care, pain management, vaccination, and diabetes treatment. Chitosan nanoparticles are shown to enhance brain delivery, improve bioavailability, and minimize systemic side effects by facilitating drug transport across the blood–brain barrier. Despite substantial advancements in targeted delivery and vaccine efficacy, challenges remain in scalability, regulatory approval, and transitioning from preclinical studies to clinical applications. The future of chitosan-based nanomedicines hinges on advancing clinical trials, fostering interdisciplinary collaboration, and innovating in nanoparticle design to overcome these hurdles and realize their therapeutic potential.

## 1. Introduction

Chitosan has been extensively explored in the field of materials science for its utility in nanostructured intranasal drug delivery systems, where researchers focus on manipulating its chemical and physical properties to enhance delivery mechanisms. Chitosan, a natural polysaccharide derived from chitin, offers several unique advantages that make it an ideal candidate for nanoparticle formulation. Its biocompatibility, biodegradability, and non-toxic nature are essential for medical applications, especially for intranasal delivery. Moreover, chitosan possesses inherent mucoadhesive properties due to its positive charge at physiological pH, which allows it to adhere to the negatively charged mucosal surfaces, enhancing the residence time and absorption of the encapsulated drugs. These attributes set chitosan apart from other polymeric nanoparticles, making it particularly suitable for targeted drug delivery and biomedical applications [1,2,3,4].

**Nanoparticle Formulation and Surface Modification**: Chitosan’s primary amine groups have been used to formulate nanoparticles with various drugs, providing a base for further chemical modifications. For example, chitosan-decorated PLGA nanoparticles have been designed to create a hybrid system that combines the biodegradability and encapsulation efficiency of PLGA with the mucoadhesive properties of chitosan [5]. Similarly, surface modification techniques involve conjugating chitosan with other molecules such as lactoferrin to target specific receptors within the nasal cavity, as seen with N-trimethylated chitosan-modified PLGA nanoparticles [6].

**Thiolation of Chitosan**: The introduction of thiol groups into chitosan molecules leads to the formation of thiolated chitosan, which shows enhanced mucoadhesiveness due to the formation of disulfide bonds with cysteine-rich subdomains of mucus glycoproteins. This modification has been utilized to develop nanoparticles for drugs like selegiline hydrochloride, aiming to improve the residence time of the formulation within the nasal cavity [7].

**Hybrid and Composite Nanoparticles**: Research has also focused on creating composite materials that combine chitosan with other biopolymers like alginate [8] or natural phospholipids such as lecithin [9]. These composites are tailored to exploit the synergistic properties of both components, such as improved encapsulation efficiency and tailored degradation rates, which are crucial for sustained release profiles.

**Coating and Layering Techniques**: Coating nanoparticles with chitosan not only provides a mucoadhesive surface but also protects the core material from premature degradation. For instance, chitosan-coated nanostructured lipid carriers [10] and chitosan-coated PLGA nanoparticles [11] demonstrate how chitosan can be used as a coating material to enhance the stability and delivery efficiency of the nanocarriers.

**Chemical Cross-linking**: To further stabilize the nanostructures and control the release of loaded agents, chitosan nanoparticles can be cross-linked using various agents. For example, the use of tripolyphosphate for ionic cross-linking of chitosan nanoparticles is a common approach to enhance their stability under physiological conditions [12].

**Water-Soluble Derivatives of Chitosan**: The native chitosan is soluble only in an acidic environment, and many pharmaceutical and biomedical application of chitosan requires the polymer to have aqueous solubility at biological pHs. N-trimethyl chitosan (TMC) enhances solubility and permeation properties across mucosal barriers, used in nanoparticles for enhanced nasal delivery [6,13,14]. Carboxymethyl chitosan (CMC), soluble and biocompatible, is used for delivering hydrophobic drugs like carbamazepine in nanoparticle form [15]. Chitosan oligosaccharides (COS), low molecular weight and highly soluble, are suitable for nasal sprays, enhancing absorption and reducing viscosity.

**Functionalization for Targeted Delivery**: Functionalization of chitosan nanoparticles with targeting ligands is another material-focused initiative. Mannose-modified chitosan nanoparticles [16], for instance, target specific receptors on nasal epithelial cells, improving the uptake and bioavailability of the encapsulated drugs.

**Dry Powder Formulations**: Addressing the challenge of delivering nanoparticles in a dry powder form for nasal administration, chitosan has been formulated into nanospheres that can be administered as dry powders, enhancing the ease of use and stability of the formulation [17].

The investigation of nanostructured chitosan for intranasal delivery is motivated by numerous challenges across various therapeutic areas, with the goal of enhancing drug delivery efficiency, increasing bioavailability, and reducing adverse effects. In the context of neurodegenerative diseases like Alzheimer’s and Parkinson’s, the primary issues are the poor bioavailability and the difficulty for therapeutic agents to cross the blood–brain barrier. Research involving chitosan nanoparticles focuses on improving the delivery to the brain of drugs such as curcumin, piperine, galantamine, huperzine A, and sitagliptin, which are typically constrained by low solubility and significant first-pass metabolism [5,6,8,18,19]. For Parkinson’s disease, studies are also examining the use of these nanoparticles to enhance the solubility and brain absorption of medications including ropinirole, bromocriptine, piribedil, and phenytoin [9,11,20,21,22].

In psychiatric care, the enhancement of central nervous system penetration and the improvement of oral bioavailability are key. Intranasal chitosan delivery systems are being developed for crucial psychiatric medications such as selegiline, risperidone, lurasidone, and olanzapine, addressing conditions like depression, schizophrenia, and bipolar disorder [7,23,24,25]. Pain management also utilizes the targeted central nervous system (CNS) delivery capabilities of nanostructured chitosan, focusing on increasing the brain delivery of pain relievers such as cyclobenzaprine and tapentadol, which aims to reduce systemic side effects [26,27].

Another major area of research is the development of nasal vaccines using chitosan nanoparticles, which seeks to boost immunogenicity and mucosal immunity against pathogens such as influenza, hepatitis, respiratory syncytial virus, pertussis, and SARS-CoV-2 [13,14,17,28,29,30,31,32,33,34,35,36,37]. These initiatives reflect the ongoing need for effective vaccination strategies that generate strong immune responses without the need for traditional injection methods.

In diabetes treatment, the exploration of intranasal chitosan delivery systems for insulin aims to enhance systemic absorption and effectiveness [38,39,40,41,42]. This method could offer a less invasive alternative to traditional subcutaneous injections, potentially improving patient adherence and overall quality of life. Moreover, the adaptability of chitosan nanoparticles is being explored in managing allergies, asthma, and nasal congestion, with formulations designed to address issues such as drug hydrophobicity, nasal irritation, and poor mucosal absorption [43,44,45].

Together, these research efforts reflect a significant and widespread interest in leveraging nanostructured chitosan for intranasal delivery as a strategy to overcome numerous challenges in drug delivery, with the ultimate goal of improving therapeutic outcomes for a variety of medical conditions.

## 2. Chitosan in Neurological Applications

### 2.1. Enhanced Brain Delivery for Neurological Treatments

#### 2.1.1. Alzheimer’s Disease

In the treatment of Alzheimer’s disease, chitosan-decorated poly(lactic-co-glycolic acid) (PLGA) nanoparticles are used to enhance the delivery of curcumin (Cur). These nanoparticles are prepared via the emulsion solvent evaporation technique and characterized using transmission electron microscopy (TEM). They improve the delivery of curcumin through the nasal mucosa to the brain, providing sustained release, enhancing permeation, and effectively scavenging reactive oxygen species [5]. Furthermore, Huperzine A (HupA) encapsulated in PLGA nanoparticles and modified with lactoferrin-conjugated N-trimethylated chitosan has been developed. These nanoparticles demonstrate enhanced mucoadhesion and targeting efficiency, which improves brain delivery, sustains release, and increases cellular uptake, showing potential for Alzheimer’s treatment [6]. Intranasal chitosan nanoparticles loaded with piperine (PIP) are designed to enhance cognitive functions and provide neuroprotection in Alzheimer’s disease. In studies with Alzheimer’s-induced rats, these nanoparticles have enhanced cognitive functions and shown significant anti-apoptotic and anti-inflammatory effects [18]. Chitosan-based nanoparticles for delivering galantamine hydrobromide (GH) have been synthesized using the ionic gelation method. Demonstrating stability over a year, these nanoparticles offer high loading efficiency and modified release profiles, which could improve delivery and minimize side effects associated with galantamine, making them suitable for nasal administration in Alzheimer’s treatment [8]. Chitosan nanoparticles also enhance the nasal absorption and brain targeting of sitagliptin for treating Alzheimer’s disease. Prepared using the ionic gelation method, these sitagliptin-loaded chitosan nanoparticles (SIT-CS-NPs) significantly increase sitagliptin levels in the brain following intranasal administration [19].

#### 2.1.2. Parkinson’s Disease

**Figure 1:**
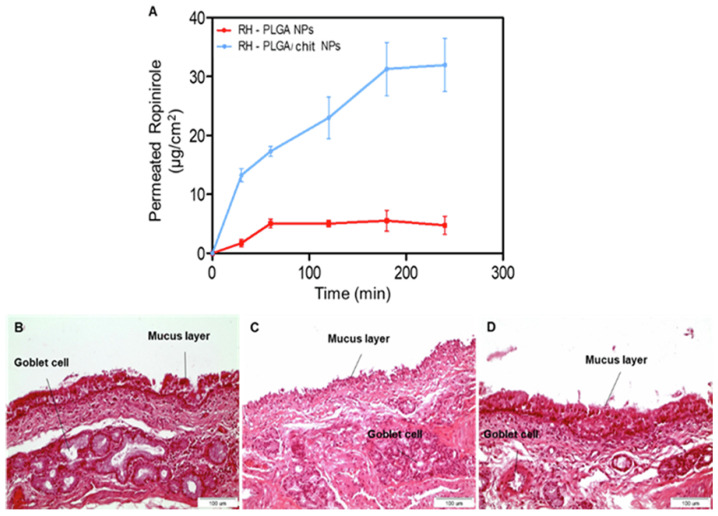
Ex vivo permeability studies of RH loaded from PLGA and PLGA/chit NPs across sheep nasal mucosa. (**A**) Permeability studies across nasal mucosa at several time points (PLGA NPs in red color, PLGA/chit NPs in blue color). Results are presented as mean value ± SEM, n ≥ 3. Histopathological studies of (**B**) untreated and (**C**) treated nasal mucosa with RH loaded PLGA NPs and (**D**) RH loaded PLGA/chit NPs after 4 h incubation. Scale bar: 100 μm, Adopted with permission [11].

Chitosan-coated poly(lactic-co-glycolic acid) (PLGA) nanoparticles have been engineered to encapsulate ropinirole hydrochloride (RH), enhancing direct CNS delivery for Parkinson’s disease treatment. These nanoparticles, prepared via the nanoprecipitation method, achieve complete drug release within 24 h and exhibit a 3.22-fold increase in permeation through the nasal mucosa compared to their uncoated counterparts, effectively delivering ropinirole directly to the CNS [11]. Figure 1 illustrates the ex vivo permeability investigations of RH loaded from PLGA and PLGA/chitosan nanoparticles (NPs) across sheep nasal mucosa.

Chitosan-coated nanostructured lipid carriers loaded with glial cell-derived neurotrophic factor (GDNF), also prepared via nanoprecipitation, demonstrated significant in vivo protection against the 6-OHDA toxin in PC-12 cells and behavioral improvements in rat models, indicating their potential for Parkinson’s disease treatment [10]. Glycol chitosan/sulfobutylether-β-cyclodextrin-based nanoparticles loaded with dopamine were prepared by crosslinking with polyanions and confirmed by techniques such as NMR and X-ray photoelectron spectroscopy. These nanoparticles have shown potential for direct brain delivery of dopamine, essential for treating Parkinson’s disease. Notably, repeated intranasal administration resulted in increased dopamine levels in the ipsilateral striatum [46]. Bromocriptine-loaded chitosan nanoparticles developed using the ionic gelation method and characterized through differential scanning calorimetry (DSC), X-ray diffraction (XRD), and scanning electron microscopy (SEM) have shown enhanced nasal permeability. These findings suggest that these nanoparticles could effectively facilitate the intranasal delivery of bromocriptine, presenting a promising method for Parkinson’s disease treatment, although further in vivo efficacy studies are required [20]. Chitosan coated intra nasal ropinirole nano emulsion was developed using sefsol 218, tween 80, transcutol, and water. The optimized nano emulsion demonstrated high translocation in the rat brain, highlighting its potential for managing Parkinson’s disease [22]. Lastly, lecithin–chitosan hybrid nanoparticles loaded with piribedil (PBD) were encapsulated within a methylcellulose thermo-responsive in situ gel, prepared using a design of experiments (DoE) approach. These nanoparticles demonstrated a significant increase in brain bioavailability in rat studies, indicating efficient and direct nose-to-brain uptake and underscoring their potential utility in treating Parkinson’s disease [9].

#### 2.1.3. Neuro-AIDS (Acquired Immunodeficiency Syndrome)

Chitosan-g-HPβCD (hydroxypropyl-β-cyclodextrin) nanoparticles containing efavirenz were developed to enhance the delivery of this antiretroviral drug to the CNS, targeting the treatment of neuro-AIDS. These nanoparticles were prepared using the ionic gelation method and optimized through quadratic response surface methodology. This formulation achieved a 12.40-fold increase in CNS bioavailability compared to the iv solution and demonstrated a 99.24% drug targeting efficiency following intranasal administration, indicating high effectiveness in targeting the CNS for neuro-AIDS treatment [47].

#### 2.1.4. Dementia and Cognitive Improvement

A chitosan-coated nanoemulgel of telmisartan was developed, comprising sefsol 218, oleic acid, tween 20, and transcutol P. The chitosan coating enhances mucoadhesive properties, improving permeation through goat nasal mucosa. This formulation has shown potential for treating dementia by facilitating enhanced delivery of telmisartan to the brain via the nasal route, suggesting significant enhancement in intranasal delivery of telmisartan [48]. Chitosan nanoparticles loaded with cyclovirobuxine D were prepared using a modified ionotropic gelation method. These nanoparticles demonstrated sustained release over 24 h and increased brain targeting via intranasal administration. The ability to increase brain concentration of cyclovirobuxine D indicates an effective brain-targeting delivery method, which could enhance treatment outcomes for cognitive impairments [12]. Lastly, polylactic acid nanoparticles were modified with chitosan to encapsulate a neurotoxin labeled with fluorescein isothiocyanate. These nanoparticles were specifically prepared and characterized for studying brain pharmacokinetics after intranasal administration in rats. The results indicated enhanced brain targeting efficiency and improved brain transport and bioavailability of the neurotoxin, suggesting potential utility in the treatment of CNS disorders through nasal delivery [49].

#### 2.1.5. Central Nervous System (CNS) Malignancies and Neuroprotection

Nanostructured lipid carriers coated with chitosan and loaded with berberine, known as BER-CTS-NLCs, were developed using hot homogenization and ultrasonication. Optimized for size, entrapment efficiency, and drug release characteristics, these carriers demonstrated enhanced delivery and pharmacokinetics within the brain, showing increased levels of the drug compared to traditional solutions, and higher brain-to-blood drug level ratios, indicating an effective strategy for CNS targeting [50]. A novel mixed amphiphilic nanoparticle system was developed using chitosan-g-Poly(methyl methacrylate) and poly(vinyl alcohol)-g-Poly(methyl methacrylate), crosslinked with sodium tripolyphosphate. Preliminary in vivo studies on mice showed increased brain bioavailability when delivered intranasally, suggesting that these nanoparticles could be effective for brain targeting and potentially reduce systemic side effects, making them viable for neurological disorder treatments [51]. Chitosan-coated nanostructured lipid carriers optimized for the delivery of protein therapies demonstrated biocompatibility and effective brain delivery following intranasal administration. The preparation and optimization for brain delivery indicate their potential for treating neurodegenerative diseases by enhancing the delivery of therapeutic agents without causing nasal mucosa toxicity [52]. Nanoparticles composed of thiolated okra gum and chitosan, prepared through an esterification process optimized using a full factorial design, exhibited promising brain-targeting properties. Their size and surface characteristics make them suitable for nasal delivery, which could be significant for non-invasive CNS drug administration [53]. Quetiapine fumarate-loaded chitosan nanoparticles (QF-NP) were optimized using ionic gelation and Box–Behnken design. These nanoparticles showed a significant brain/blood ratio and nasal bioavailability, indicating enhanced brain delivery via the intranasal route. The high encapsulation efficiency and significant brain targeting highlight the potential of this delivery method for reducing dosage and decreasing side effects associated with oral administration [54]. Temozolomide-loaded nano lipid chitosan hydrogel (TMZNLCHG), prepared using a blend of chitosan and Vitamin E with gelucire 44/14, demonstrated enhanced nasal absorption and compatibility with glioma cell lines. Aimed at targeting the brain for treating metastatic melanoma and glioma, this formulation leads to higher bioavailability and less nasal mucosa damage, indicating its potential for improving glioblastoma treatment outcomes [55]. Mucoadhesive chitosan-coated nanoemulsions optimized for the nasal delivery of rosmarinic acid using the Box–Behnken design exhibited high mucoadhesive potential and superior penetration/retention capabilities through the nasal mucosa. These nanoemulsions could significantly improve the delivery of rosmarinic acid for neuroprotective applications [56]. HP-β-CD/chitosan nanoparticles developed to load scutellarin for intranasal delivery were designed to enhance brain delivery, particularly for treating cerebral ischemia. These nanoparticles showed increased brain accumulation compared to other administration routes, suggesting significant potential for this delivery method [57]. Intranasal galantamine/chitosan complex nanoparticles, formulated to enhance neuroprotective effects through antioxidant mechanisms, showed no biochemical toxicity in rat brains. They effectively reduced oxidative stress markers while improving antioxidant levels, supporting their potential for neuroprotection [58]. Lastly, simvastatin-loaded poly-epsilon-caprolactone nanocapsules, coated with chitosan of varying molecular weights, displayed high encapsulation efficiency and controlled drug release. Prepared using a novel one-pot technique, these nanocapsules also showed enhanced nasal permeation of simvastatin, indicating their potential utility in treating CNS malignancies by facilitating enhanced brain delivery [59].

### 2.2. Targeting Specific Neurological Conditions

#### 2.2.1. Epilepsy and Other Specific Neurological Conditions

Bioengineered PLGA–chitosan nanoparticles were developed to load thyrotropin-releasing hormone analogues, NP-355 and NP-647, aimed at the brain-targeted intranasal delivery of antiepileptic drugs. These nanoparticles were evaluated for their physicochemical properties, sustained release capabilities, and antiepileptic potential following intranasal administration. The studies demonstrated successful brain delivery and sustained release of the compounds, indicating their potential in the treatment of epilepsy [60]. Chitosan–lecithin nanoparticles loaded with phenytoin (PHT) were prepared via the nano-precipitation method to optimize brain delivery. These nanoparticles showed optimal preparation characteristics, including sustained release and increased brain levels of PHT when administered intranasally. This enhanced brain targeting and sustained release of phenytoin demonstrate the potential of intranasal delivery of PHT-loaded nanoparticles for managing epilepsy [21]. Carboxymethyl chitosan nanoparticles loaded with carbamazepine were prepared specifically for enhanced brain delivery via the intranasal route to treat epilepsy. The small particle size, high drug loading, and high entrapment efficiency contribute to increased bioavailability and effective brain targeting, suggesting an improved approach for enhancing drug concentration and treatment efficacy in epilepsy [15].

#### 2.2.2. General and Specific CNS Targeting Strategies

Chitosan-coated PLGA nanoparticles designed for the nasal delivery of carmustine were prepared using the double emulsion solvent evaporation method. These nanoparticles demonstrated enhanced brain permeability and an increased area under the curve (AUC) in the brain compared to both intranasal and intravenous solutions of carmustine. This enhanced permeability and sustained release facilitate a high therapeutic efficacy of carmustine in the treatment of glioblastoma [61]. Curcumin-laden dual-targeting nanocarriers, composed of chitosan and fucoidan, were developed to enhance brain delivery via the intranasal route, specifically targeting inflammatory brain lesions. This targeted delivery system shows enhanced drug delivery, distribution, and accumulation in brain lesions, augmenting the therapeutic effects against brain inflammation [62]. Chitosan-coated buspirone-loaded nanostructured lipid carriers (BPE-CH-NLCs) were prepared using the solvent diffusion evaporation technique and optimized using a quality by design (QbD) approach with the Box–Behnken design. These nanoparticles showed high drug-targeting efficiency and a high direct transport percentage to the brain, confirming their potential for intranasal delivery as an effective brain-targeting strategy [63]. Selegiline hydrochloride-loaded thiolated chitosan nanoparticles were developed using the ionic gelation method to enhance nasal delivery for the treatment of depression. The thiolated chitosan nanoparticles have improved the nasal delivery and bioavailability of selegiline, evidenced by behavioral tests that suggest antidepressant activity [7]. A nanocomposite of thiolated chitosan and Centella asiatica, formulated for brain targeting via the nasal route, was characterized using Fourier-transform infrared spectroscopy (FTIR), X-ray diffraction (XRD), nuclear magnetic resonance (NMR), and mass spectrometry (MS). The nanoparticles were formed via ionic gelation. This enhanced delivery of Centella to the CNS could potentially treat various neurological disorders [64]. Chitosan-siRNA nanoparticles formulated for intranasal delivery target the brain as a non-invasive method to bypass the blood–brain barrier, utilizing the olfactory and trigeminal pathways. This method has demonstrated its efficiency and safety in brain drug delivery, making it a viable option for neurodegenerative disease treatment [65]. Chitosan–mangafodipir nanoparticles have been designed for the intranasal delivery of siRNA and DNA directly to the brain. This innovative delivery system has proven effective following intranasal administration, demonstrating the potential of this system for non-invasive gene therapy delivery in treating neurodegenerative diseases [66]. Enriched chitosan nanoparticles loaded with anti-HTT siRNA have been developed for the treatment of Huntington’s disease. In the YAC128 mouse model, these nanoparticles effectively targeted and reduced the expression of the mutant HTT gene, highlighting the potential of intranasal siRNA delivery as a non-invasive therapeutic alternative for chronic brain disease treatment [67].

Table 1 provides an overview of various chitosan-based nanoparticle formulations utilized for brain targeting and drug delivery applications. From intranasal delivery to enhanced mucoadhesion and sustained release, these formulations showcase the versatility of chitosan in facilitating efficient drug delivery to the brain for various therapeutic purposes.

## 3. Immunization and Immune Response Enhancement

### 3.1. General Vaccine Delivery Improvements

#### 3.1.1. Influenza, Hepatitis B, and Other Respiratory/Mucosal Vaccines

Influenza Vaccines: N-trimethyl chitosan (TMC) nanoparticles loaded with the influenza A subunit H3N2 antigen were prepared by combining TMC and antigen with tripolyphosphate (TPP) at a pH of 7.4 and ambient temperature. This formulation maintained the integrity of the antigen and induced high immune responses following intranasal administration. It generated stronger hemagglutination inhibition and IgG responses than those observed with traditional intramuscular antigen administration, suggesting that nasal delivery of this vaccine could be more effective for inducing immune protection against influenza [13]. Another application of N-trimethyl chitosan (TMC) involved nanoparticles encapsulating the r4M2e.HSP70c antigen for nasal vaccination. This formulation enhanced both the longevity and levels of M2e-specific IgG antibodies, induced Th1-skewed humoral and cellular responses, and offered full protection against a lethal influenza dose in mice, demonstrating its effectiveness in provoking robust immune responses and protection via intranasal delivery [14]. Influenza A (H1N1) antigen was covalently conjugated to N-trimethylaminoethylmethacrylate chitosan (TMC) nanoparticles through thioester bonds. SDS-PAGE analysis confirmed that most of the antigen was successfully conjugated to the nanoparticles, maintaining stability, and enhancing immunogenicity. After nasal administration, there was a significant increase in both serum IgG and mucosal sIgA levels, along with improved cytokine production, enhancing the immunogenicity of the H1N1 antigen following nasal administration, and demonstrating its effectiveness as a nasal vaccine against H1N1 [29]. Chitosan nanospheres containing influenza virus and adjuvants (CpG oligodeoxynucleotide (CpG ODN) and Quillaja saponin) were prepared using ionic gelation with tripolyphosphate. These nanospheres showed a controlled release of antigen and adjuvants and exhibited only slight cytotoxicity, which was further reduced by co-encapsulation with adjuvants, making this approach a promising method for nasal influenza vaccine delivery [28]. A dry powder form of chitosan nanospheres encapsulated with influenza whole virus and adjuvants (CpG oligodeoxynucleotide (CpG ODN) and Quillaja saponins) was nasally administered to rabbits, significantly enhancing both humoral and cellular immune responses and inducing significant cytokine production. This formulation demonstrated effective mucosal immunization against influenza, showing a significant elevation in hemagglutination inhibition (HI) antibody titers and serum IgG levels, particularly with the CpG adjuvant, suggesting an effective strategy for nasal immunization [17].

Hepatitis B Vaccines: Glycol chitosan (GC) nanoparticles designed for the intranasal delivery of the hepatitis B vaccine have been shown to induce strong systemic and mucosal immune responses, significantly outperforming traditional chitosan nanoparticles. This makes glycol chitosan nanoparticles a promising platform for mucosal vaccine delivery [32]. N,N,N-trimethyl chitosan (N-TMC) nanoparticles, loaded with the hepatitis B virus surface antigen (HBsAg), demonstrated enhanced stability and adjuvanticity in vivo, providing a stable and efficient delivery system for the HBV surface antigen. This controlled release over an extended period and the in vivo stability of the nanoparticles show promise for their use in intranasal vaccination against hepatitis B [33]. Alginate-coated chitosan nanoparticles loaded with the hepatitis B surface antigen (HBsAg) and co-delivered with CpG oligodeoxynucleotide (ODN) have been shown to induce humoral mucosal immune responses. When combined with CpG ODN, these nanoparticles also generated a Th1-biased systemic immune response and higher interferon-gamma production compared to administering recombinant hepatitis B surface antigen (HBsAg) alone. This indicates that the nanoparticles can generate a Type 1 T helper (Th1)-based immune response and higher interferon-gamma production, highlighting their effectiveness as a vaccination strategy against hepatitis B [68]. Chitosan nanoparticles loaded with DNA and human serum albumin (HSA) for hepatitis B surface antigen (HBV) vaccination demonstrated that HSA-CH NP (chitosan nanoparticles)/DNA complexes elicited robust systemic immune responses and specific IgA at mucosal sites. This formulation showed potential as an effective gene delivery system for nasal vaccination against HBV, indicating that intranasal immunization with these nanoparticles induced HBV-specific IgA in secretions and a robust systemic immune response [30].

**Figure 2:**
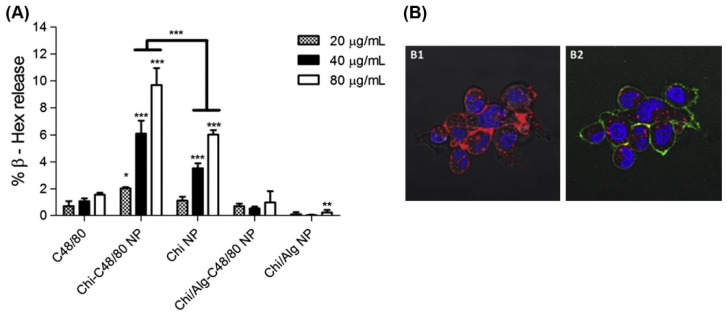
Evaluation of mast cell activation by nanoparticles. (**A**) Degranulation of the mast cell line HMC-1 was evaluated by a β-hex release assay. Cells were stimulated with C48/80 at 20, 40 or 80 μg/mL in solution or incorporated in nanoparticles. Blank Chi NP and Chi/Alg NP were used as controls at the same concentration of nanoparticles tested with C48/80 loaded particles. Not only Chi-C48/80 NP but also Chi NP induced a higher % of β-hex release than C48/80 in solution and Chi/Alg-C48/80 NP, indicating the intrinsic ability of Chi NP to activate mast cells. Data are representative of three independent experiments performed in triplicate or quadruplicate, mean ± SD, n = 3. Symbols above bars indicate the differences relative to C48/80 in solution, * *p* < 0.05, ** *p* < 0.01, *** *p* < 0.001 2-way ANOVA (**B**) Confocal images of HMC-1 before (**B1**) and 2 min after treatment with FITC labeled Chi-C48/80 NP (green) at a dose corresponding to 40 μg/mL of C48/80 in Tyrode’s solution (**B2**). Cells were labeled with Hoechst 33,342 (blue) for nuclei and with Alexa Fluor^®^ 594 WGA to identify cell membranes. Images showed that Chi-C48/80 immediately adsorbed on the cell surface, Adopted with permission [69].

Respiratory and General Mucosal Vaccines: Chitosan and chitosan–alginate nanoparticles loaded with a mast cell activator compound 48/80 (Chi-C48/80 NP and Chi/Alg-C48/80 NP) have been developed. Among these, the Chi-C48/80 NP demonstrated superior adjuvant effects, including greater mast cell activation, enhanced cellular uptake, and improved nasal residence time of the model antigen compared to the Chi/Alg-C48/80 NP. This enhanced performance results in better mucosal and systemic immune responses when administered via the nasal route. Specifically, the Chi-C48/80 NP exhibited superior adjuvant properties, more effective mast cell activation, and induced higher serum antibody levels compared to other formulations, making it a more effective candidate for nasal vaccine delivery [69]. Figure 2 illustrates the activation of mast cells by chitosan nanoparticles and inducing β-hexosaminidase release. N-trimethyl chitosan (TMC) nanoparticles encapsulating Bordetella pertussis antigens were found to induce strong immune responses (IL-4, IL-17, IFN-gamma, IgG, IgA) in mice, suggesting their potential for effective systemic and mucosal immunity against pertussis, particularly suitable for preventing respiratory tract transmission. These nanoparticles effectively induced a range of immune responses, suggesting improved efficacy in pertussis prevention by targeting both systemic and local mucosal immune systems [31]. Curdlan sulfate–O-linked quaternized chitosan nanoparticles were specifically designed to improve the immunogenicity of antigens through intranasal administration. These nanoparticles facilitate antigen uptake by epithelial cells and significantly enhance immune responses, more so than traditional adjuvants. The enhanced uptake of antigens and activation of immune cells by these nanoparticles leads to higher levels of specific antibodies, suggesting their effectiveness as nasal vaccine adjuvants [70].

#### 3.1.2. Enhancements in Nasal Vaccine Delivery

Vaccines for other diseases and their applications with chitosan: Low-molecular-weight chitosan nanoparticles, serving as nasal vaccine delivery vehicles for tetanus toxoid (TT), induced significant long-lasting humoral and mucosal immune responses in mice, demonstrating the potential utility of low molecular weight chitosan for nasal immunization [71]. Quaternized chitosan nanoparticles using N-2-hydroxypropyl trimethyl ammonium chloride chitosan (N-2-HACC) and N,O-carboxymethyl chitosan (CMC) as carriers loaded with a combined live vaccine against Newcastle disease (ND) and infectious bronchitis (IB) induced higher IgG and IgA titers, lymphocyte proliferation, and cytokine levels in chickens than those achieved with commercial vaccines, indicating the potential of these nanoparticles as effective mucosal vaccine carriers, particularly for poultry [72].

### 3.2. Immune Enhancement for Specific Diseases

#### 3.2.1. Comparative and Mechanistic Studies of Chitosan-Based Formulations

A comparative study revealed that TMC nanoparticles (TMC NPs), which rapidly released antigens and induced dendritic cell maturation, were superior for nasal immunization. These nanoparticles, due to their positively charged nature, induced high serum antibody titers and mucosal Immunoglobulin A levels, suggesting their suitability for nasal vaccination through improved nasal residence time and stimulation of dendritic cells [73]. N-trimethylaminoethylmethacrylate chitosan (TMC) nanoparticles with surface-conjugated antigens have shown enhanced cellular uptake and higher levels of systemic and mucosal immune responses following nasal administration. The transport mechanism suggested efficient transport across the nasal epithelium and uptake in nasopharynx-associated lymphoid tissue, leading to strong immune responses with no observed toxicity to the nasal mucosa, demonstrating a targeted delivery approach that significantly enhances both systemic and mucosal immune responses [74]. Chitosan–dextran sulfate nanoparticles loaded with pertussis toxin (PTX) and IgA showed potential as a nasal vaccine delivery system. Significant in vivo uptake, likely via nasal membranous or microfold cells, was observed, suggesting that IgA-loaded nanoparticles can be preferentially taken up by nasal cells, highlighting their efficiency as a delivery system for inducing a targeted immune response [75]. Recombinant Staphylococcus aureus enterotoxin B (rSEB) was loaded into chitosan nanoparticles synthesized using ionic gelation. Comparative immunogenicity studies between the rSEB-loaded nanoparticles and the bare rSEB formulation in mice showed that both evoked immune responses without significant differences, suggesting that the nano-delivery system did not notably enhance immunogenicity over the bare rSEB, crucial for understanding the effectiveness of nanoparticle systems in vaccine development [76]. Alginate-coated chitosan and trimethyl chitosan nanoparticles loaded with PR8 influenza virus were prepared by directly coating the virus with polymers. This formulation induced stronger immune responses than uncoated formulations, demonstrating its potential as an effective nasal vaccine delivery system [77]. A study examining the adjuvanticity of N,N,N-trimethyl chitosan (TMC) with varying degrees of acetylation in an intranasal influenza virus vaccine found that higher acetylation levels reduced adjuvant effects, possibly due to decreased stability in the nasal cavity. This investigation elucidates the critical factors influencing the adjuvanticity of TMC in nasal vaccines, indicating that while higher acetylation may slightly diminish stability, it does not adversely affect the overall immune response [78]. Table 2 summarizes the diverse applications of chitosan-based nanoparticles in nasal vaccination against infectious diseases. From influenza antigens to hepatitis B vaccines, these formulations demonstrate promising outcomes in inducing strong systemic and mucosal immune responses. The table includes details such as the type of chitosan used, encapsulated antigens, and the observed immune responses, along with corresponding references.

#### 3.2.2. Broad-Spectrum and Disease-Specific Immune Responses

**Figure 3:**
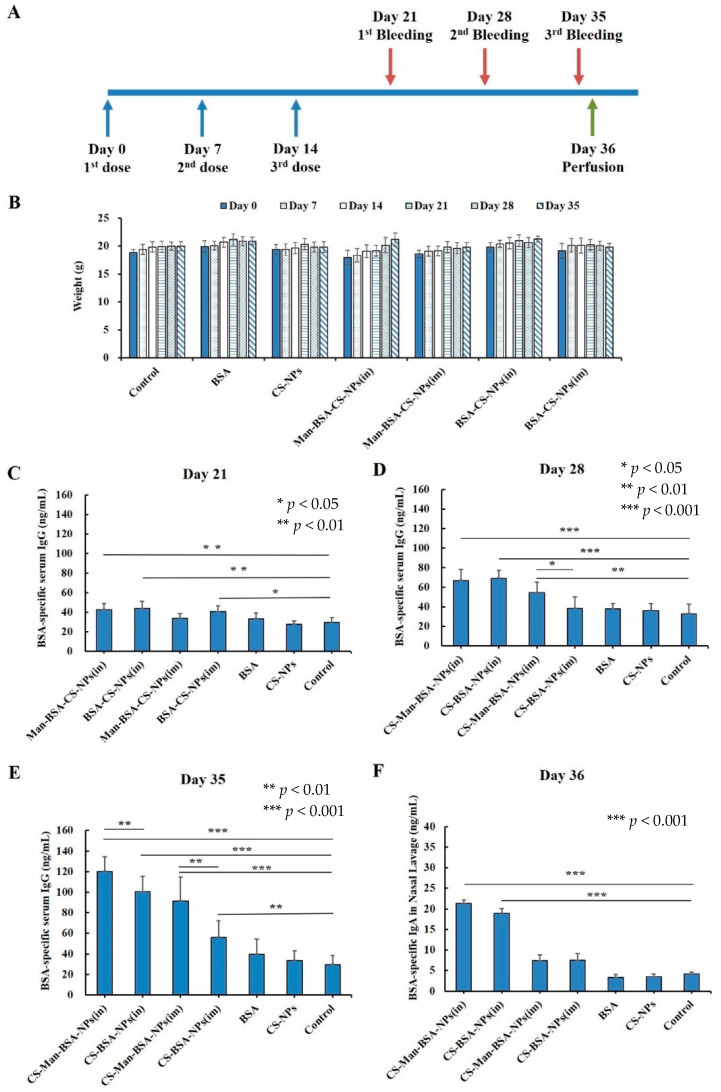
Immunization and sampling timeline (**A**); changes in body weight of mice (**B**); BSA-specific serum IgG (**C**–**E**) and nasal lavage fluid (NLF) IgA (**F**) antibody responses; all results are reported as mean (SD) (n = 6, * *p* < 0.05, ** *p* < 0.01, *** *p* < 0.001) [16].

General Improvements in Nasal Vaccine Delivery: Protein-loaded N-trimethyl chitosan nanoparticles have been developed using ionic crosslinking with tripolyphosphate. They effectively preserve protein integrity and control release, exhibiting no toxicity in tests assessing ciliary function and cell viability. The ability of these nanoparticles to transport protein through the nasal mucosa was particularly notable, with over 70% of the protein remaining associated with the nanoparticles for at least three hours, underscoring their potential for nasal delivery of proteins [79]. N-trimethyl chitosan nanoparticles, containing ovalbumin along with various immunopotentiators, were specifically tailored to enhance the immunogenicity of subunit antigens via both nasal and intradermal routes. Nanoparticles with ligands lipopolysaccharide (LPS) or muramyl dipeptide (MDP) showed heightened immune responses following nasal administration, highlighting the critical role of adjuvant choice and administration route on their effectiveness [80]. Mannose-modified chitosan nanoparticles loaded with bovine serum albumin (BSA) as a model antigen have been developed. In vivo studies indicated significant enhancements in both mucosal and systemic immune responses, suggesting that these mannose-modified chitosan nanoparticles are a promising system for intranasal vaccine delivery. They effectively enhanced antigen adhesion and absorption, leading to increased specific IgG and IgA responses (Figure 3) [16].

Ovalbumin-loaded N-trimethyl chitosan (TMC) nanoparticles combined with CpG DNA (TMC/CpG/OVA) were prepared by substituting unmethylated CpG DNA for tripolyphosphate (TPP) to enhance Th1 responses. These nanoparticles induced stronger Th1 responses, evidenced by higher IgG2a levels and increased T-cell activation, proving the effectiveness of these nanoparticles as a nasal vaccine delivery system. The use of CpG DNA significantly enhanced the immune response towards a Th1 bias, characterized by an increased IgG2a response and greater numbers of Interferon (IFN)-gamma-producing T-cells [81]. Covalently stabilized trimethyl chitosan-hyaluronic acid nanoparticles loaded with ovalbumin (OVA) were prepared using ionic gelation followed by disulfide bond formation and further stabilized with PEGylation. Despite PEGylation slightly reducing nasal immunogenicity, the stabilized nanoparticles exhibited enhanced physical stability and immunogenicity, leading to higher IgG titers compared to non-stabilized particles [82]. Chitosan-DNA nanoparticles expressing the pneumococcal surface antigen A (PsaA) were developed for intranasal immunization against Streptococcus pneumoniae. This formulation effectively reduced pneumococcal nasopharyngeal colonization, underscoring its potential as a robust immunization strategy against Streptococcus pneumoniae [83]. Chitosan/pCETP nanoparticles, formulated with chitosan and a DNA plasmid encoding a B-cell epitope of cholesteryl ester transfer protein, were used for intranasal immunization against atherosclerosis in cholesterol-fed rabbits. The results indicated a strong systemic immune response and highlighted the potential of this non-invasive vaccine delivery method for combating atherosclerosis, with sustained anti-CETP (cholesteryl ester transfer protein) IgG titers and notably lower aortic lesion areas [84].

##### Respiratory Pathogens Immune Enhancement via Intranasal Delivery

Chitosan–pullulan composite nanoparticles have been developed for the nasal delivery of vaccines, utilizing bovine serum albumin (BSA) as a model antigen. These nanoparticles demonstrated efficient cellular uptake and lower toxicity, underscoring their suitability for nasal vaccination [85]. Chitosan–pullulan composite nanoparticles have also been specifically developed for delivering diphtheria toxoids via nasal routes. Immunological assessments have indicated that these nanoparticles induce higher levels of DT-specific IgG when delivered nasally compared to traditional intramuscular administration. The co-encapsulation of CpG ODN within these nanoparticles has enhanced the Th-1/Th-2 immune response, solidifying their potential as an effective nasal vaccine delivery system [86]. Functionalized chitosan-based composite nanoparticles, specifically N-2-hydroxypropyl trimethyl ammonium chloride chitosan/N,O-carboxymethyl chitosan (N-2-HACC/CMCS) nanoparticles, were developed for intranasal delivery. They exhibited excellent biostability, mucosal absorption, and immune activation capabilities, excelling in promoting lymphocyte proliferation and pro-inflammatory factor secretion, crucial for stimulating specific immune responses [87]. Mixed formulations comprising cross-linked dextran microspheres and tetanus toxoid-loaded trimethyl chitosan nanospheres have been used for nasal delivery in a dry powder form. This combination significantly boosted both systemic IgG and mucosal sIgA responses in a rabbit model, showcasing the potent adjuvant capabilities of the mixture [88]. Plasmid-DNA-loaded chitosan nanoparticles have been formulated for hepatitis B nasal mucosal immunization. Nasal administration of these nanoparticles induced protective immunoglobulin levels and specific cellular responses, demonstrating the potential of chitosan nanoparticles as effective DNA vaccine carriers for non-invasive nasal immunization routes [34]. Dendritic-cell-targeted chitosan nanoparticles, biotinylated and loaded with plasmid DNA, targeted dendritic cells through a receptor-mediated gene delivery approach specifically designed for nasal immunization against SARS-CoV. The results showed significant mucosal IgA and systemic IgG responses against the nucleocapsid protein, demonstrating the potential for effective low-dose nasal vaccines [89]. Mannosylated chitosan nanoparticles for nasal mucosal delivery were developed to encapsulate a DNA vaccine, showing enhanced delivery efficiency and significant tumor immunity in a mouse model. These nanoparticles are efficiently bound to C-type lectin receptors on macrophages, enhancing both cellular and humoral immune responses against tumors. This approach showcased their potential for antitumor immunotherapy and nasal mucosal delivery [90]. Chitosan nanoparticles loaded with Newcastle disease virus DNA vaccine have been developed, demonstrating enhanced mucosal, humoral, and cellular immune responses in chickens, indicating superior efficacy in intranasal immunization compared to intramuscular delivery [91].

##### Gastrointestinal and Enteric Pathogens Immune Enhancement

Bacterial Infections: Chitosan nanoparticles loaded with B. abortus malate dehydrogenase induced pro-inflammatory cytokine production and systemic Immunoglobulin A (IgA) in mice through intranasal administration, showing potential for robust immune responses, particularly in combating Brucella abortus infections. These nanoparticles activated immune cells and pathways related to mucosal immunity in the nasal-associated lymphoid tissue (NALT), leading to significant production of specific IgA [92]. Chitosan-DNA nanoparticles administered intranasally to chickens produced high levels of systemic IgG and mucosal IgA, effective in reducing bacterial colonization, suggesting their potential for preventing Campylobacter infections in poultry. This prophylactic strategy is effective in increasing serum and mucosal antibody levels, consequently reducing bacterial expulsion and potentially curbing the spread of infection [93]. Electrospun chitosan nanofibrous membranes administered intranasally to guinea pigs induced significant serum and mucosal antibody responses and provided protection against *S. flexneri* challenge, highlighting their effectiveness as carriers for nasal vaccines. Guinea pigs vaccinated nasally exhibited higher serum and mucosal antibody responses and enhanced protection against Shigella infection [94]. The polyglutamic acid-trimethyl chitosan peptide nano-vaccine induced potent systemic and mucosal antibody responses against Group A Streptococcus (GAS) in mice, outperforming standard adjuvants. Moreover, this delivery system effectively reduced the bacterial burden in challenge studies, illustrating its potential for enhancing peptide-based vaccine delivery [95]. Chitosan-based nanoparticles for Japanese encephalitis demonstrated enhanced mucosal immune responses and higher levels of secretory IgA (sIgA) compared to traditional subcutaneous immunization. These results show the potential of this non-invasive vaccination route to enhance both mucosal and systemic immunity against Japanese encephalitis [96]. Alginate–chitosan nanoparticles for tuberculosis vaccine induced strong Th1-biased immune responses and notable IL-17 cytokine elicitation, demonstrating effectiveness as both a booster strategy for subcutaneous vaccines and as a prime strategy through intranasal vaccination. This dual approach enhances the efficacy of Bacille Calmette–Guérin (BCG) vaccines and induces strong immune responses, showing the potential to improve BCG vaccine efficacy for tuberculosis [97].

Viral Infections: Chitosan–alginate nanoparticles encapsulated with bee venom were intranasally delivered to pigs to enhance T-cell responses and aid in viral clearance in cases of porcine reproductive and respiratory syndrome virus (PRRSV). This treatment bolstered systemic immune responses, particularly Type 1 T helper (Th1) responses, effectively reduced viral load, and mitigated immune suppression. The pigs showed only mild signs of pneumonia, coupled with a marked decrease in immune-suppressive actions, suggesting these nanoparticles could be a viable treatment option for PRRSV infections [98]. Chitosan nanoparticles for swine influenza vaccine encapsulated an inactivated influenza vaccine administered intranasally to pigs. This approach provided cross-reactive protection against various influenza strains, decreased viral shedding, and enhanced cellular immune responses, outperforming controls. The vaccination led to increased IgG and mucosal IgA, reduced viral shedding, and lowered lung virus titers, demonstrating effective immunity against swine influenza A virus (SwIAV) [99].

Fungal Infections: Chitosan nanoparticles encapsulating the P10 peptide from Paracoccidioides brasiliensis were confirmed as non-toxic through hemolytic tests and cell viability assays on murine macrophages. Intranasal immunization with these nanoparticles led to a reduction in fungal load and the standard peptide concentration needed, signifying effective immune modulation. The intranasal vaccine employing P10 peptide–chitosan nanoparticles elicited a robust Th1-type immune response and significantly lowered the fungal burden in a murine model of Para coccidioidomycosis (PCM), marking it as a promising therapeutic option [100]. Chitosan nanoparticles with fluorescent labeling, carrying the P10 peptide, primarily localized in the upper airway, with smaller quantities reaching the trachea and lungs. These nanoparticles effectively reduced the fungal load and the number of doses required for an effect, while inducing Th1 and Th17 immune responses. This intranasal vaccine based on chitosan nanoparticles successfully triggered potent immune responses and significantly reduced the fungal burden with fewer doses needed, affirming its potential as a therapeutic vaccine against PCM [101].

Other Pathogens: Chitosan nanoparticles with inactivated Chlamydia psittaci EBs induced higher levels of IFN-gamma in chickens and showed enhanced pharyngeal bacterial clearance and protection against lung lesions compared to cytidine-guanosine-dinucleotides (CpG) adjuvanted groups. The study suggests a dose-dependent protective immunity with varying concentrations of VCG, demonstrating effective induction of protective immunity and highlighting the potential of chitosan nanoparticles as delivery systems for mucosal vaccines [102].

Table 3 showcases the diverse applications of chitosan nanoparticles in vaccine development, particularly focusing on nasal delivery. From various protein antigens to DNA vaccines, these formulations demonstrate enhanced mucosal and systemic immune responses.

## 4. Systemic Delivery and Drug Absorption

### 4.1. Applications in Disease Treatments

#### 4.1.1. Neurological and Psychiatric Disorders

Chitosan nanoparticles have been employed to enhance the delivery and bioavailability of galantamine, a drug used in the treatment of Alzheimer’s disease, within the rat brain and plasma. Utilizing a validated liquid chromatography-mass spectrometry (LC-MS) method for bioanalysis, these nanoparticles have improved brain uptake and pharmacokinetics of galantamine, surpassing both oral and nasal delivery methods. This indicates their capability for targeted delivery, potentially optimizing the therapeutic effects of galantamine in Alzheimer’s disease models [103]. Olanzapine-loaded chitosan nanoparticles, administered intranasally in rabbits, have improved both the systemic absorption and absolute bioavailability of olanzapine compared to intranasal solutions. This underscores the potential of chitosan nanoparticles as an effective delivery system to enhance the systemic availability of psychiatric medications, suggesting a promising approach for drug delivery that could improve therapeutic outcomes in psychiatric disorder treatments [23]. Dihydroergotamine-loaded chitosan nanoparticles, also administered intranasally to rats, significantly increased the bioavailability of dihydroergotamine over standard intranasal solutions. This increase in bioavailability is particularly promising for the acute treatment of migraines, suggesting that chitosan nanoparticles could serve as an efficient delivery mechanism for quick migraine relief [104].

#### 4.1.2. Infectious Diseases and Antimicrobial Applications

Didanosine-loaded chitosan nanoparticles administered intranasally in rats led to higher concentrations of didanosine in the blood, cerebrospinal fluid (CSF), and brain tissues than those achieved with intravenous administration. These findings indicate that intranasal delivery of didanosine through chitosan nanoparticles enhances both systemic and brain targeting of the drug, potentially offering substantial benefits for treating infections caused by AIDS viruses. This enhanced delivery method could lead to increased drug efficacy and improved outcomes for patients with AIDS [105]. Zidovudine-loaded chitosan nanoparticles, developed in two formulations differentiated by the chitosan-to-TPP ratio (NP1 and NP2), demonstrated improved mucoadhesion and drug flux across the nasal mucosa. This suggests their potential to enhance the therapeutic efficacy of zidovudine for AIDS treatment via nasal delivery [106]. A hydrogel nanocomposite consisting of silver nanoparticles, chitosan, PVA, and PEG was developed for application on cotton nasal tampons. Demonstrating good water retention, blood coagulation capabilities, and antibacterial activity against E. coli and S. aureus, this hydrogel shows potential for medical use in nasal tampons, where it can provide effective antimicrobial activity and enhanced physical properties [107].

#### 4.1.3. Insulin and Diabetes Management

**Figure 4:**
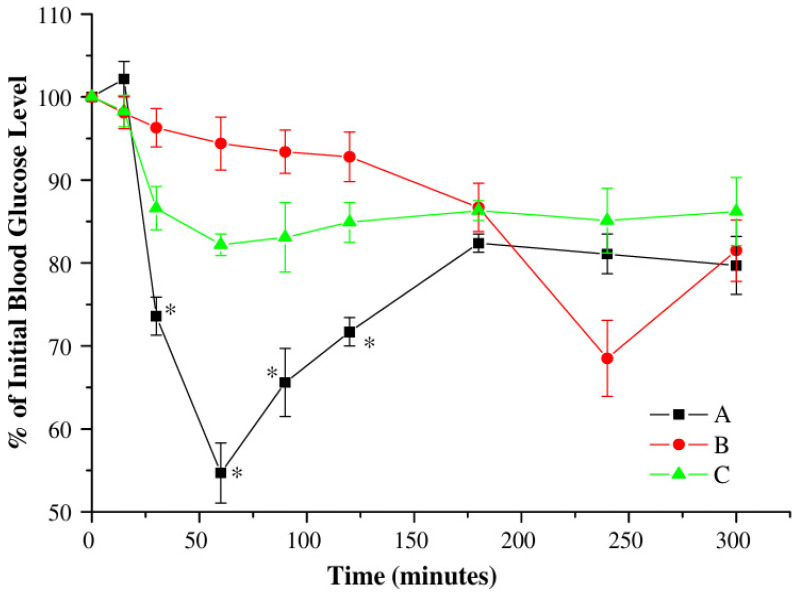
Plasma glucose levels in rabbits following nasal administration (at pH 7.4) of: A, insulin-loaded PE3gC24 nanoparticles suspended in PBS; B, insulin-PE3gC24 PBS suspension; C, insulin in PBS. Mean ± SD, n = 6. * Statistically significant differences from control insulin solution (*p* < 0.05), Adopted with permission [42].

Chitosan nanoparticles for insulin delivery were assessed by comparing the nasal absorption of insulin in various forms: chitosan nanoparticles, chitosan solution, and chitosan powder. Although the nanoparticles induced a pharmacological response, the chitosan powder exhibited higher bioavailability and was more effective for nasal insulin delivery in animal models, suggesting that chitosan powder might be more advantageous for nasal insulin delivery [38]. Freeze-dried insulin-loaded chitosan nanoparticles were developed for nasal administration and characterized by their stability post-reconstitution. In vivo tests on rabbits showed a notable reduction in glucose levels, confirming that these nanoparticles can effectively deliver insulin nasally and could provide a feasible option for diabetes management [39]. Chitosan nanoparticles for nasal insulin delivery involved creating insulin-loaded nanoparticles through ionotropic gelation. These nanoparticles significantly enhanced the systemic absorption of insulin compared to chitosan solution alone, establishing their utility as an effective nasal delivery system for insulin [40]. trimethyl chitosan and PEGylated nanocomplexes for insulin explored the nasal absorption of insulin using nanocomplexes made from trimethyl chitosan (TMC) or PEGylated TMC. The effect on the nasal mucosa varied based on the charge ratio of insulin to polymer, with the PEGylated TMC showing reduced toxicity. This finding points to the potential of these formulations to enhance nasal insulin absorption while minimizing toxicity to the nasal epithelium [108]. Chitosan-N-acetyl-L-cysteine nanoparticles for Insulin were synthesized by in situ gelation with tripolyphosphate (TPP). They displayed rapid swelling in solution and exhibited a burst followed by controlled insulin release, effectively enhancing insulin absorption through the nasal mucosa [41]. PEG-grafted chitosan nanoparticles for insulin, prepared by ionotropic gelation of polyethylene glycol-grafted chitosan with tripolyphosphate ions, demonstrated superior nasal absorption of insulin in rabbits compared to other insulin formulations. The enhanced systemic absorption following nasal administration underscores the potential of these nanoparticles as a non-invasive delivery system for peptide drugs [42]. Figure 4 depicts the in vivo study results showing the plasma glucose levels of rabbits after intranasal administration of insulin-PE3gC24 suspension, insulin-loaded PE3gC24 nanoparticles, and the control insulin solution.

#### 4.1.4. Cardiovascular and Blood Pressure Management

Chitosan lactate nanoparticles for enalaprilat were created using sodium tripolyphosphate to cross-link chitosan lactate, encapsulating enalaprilat through ionic gelation. These nanoparticles demonstrated prolonged nasal drug permeation and were effective in controlling blood pressure in rats. Nasal toxicity studies on sheep mucosa further confirmed the safety of this formulation, indicating that the nanoparticles maintain stable morphology, provide sustained drug release, and effectively manage blood pressure without causing nasal toxicity [109]. Chitosan nanoparticles for olmesartan medoxomil were formulated using the ionotropic gelation method. These nanoparticles efficiently permeated the nasal mucosa, and pharmacokinetic studies indicated a significant increase in bioavailability compared to oral suspension. They also proved effective in reducing blood pressure and heart rate in hypertensive rats without damaging the nasal mucosa, suggesting enhanced systemic delivery of olmesartan for hypertension treatment via the nasal route [110].

#### 4.1.5. Allergic Rhinitis and Airway Inflammatory Diseases

Adaptive chitosan-based nano-vehicles for cetirizine, such as deoxycholate–chitosan-hydroxybutyl nanoparticles encapsulating cetirizine, were synthesized to exhibit pH-responsive and temperature-sensitive drug release behaviors. These properties are suitable for nasal conditions, allowing the particles to swell under enzymatic conditions, which suggests potential for responsive release in treatments for allergic airway inflammatory diseases. The nanoparticles adapted well to nasal conditions, offering effective and less irritating delivery of cetirizine for allergic rhinitis treatment. Their pH-responsive and thermo-sensitive properties facilitated controlled drug release, making them ideal for nasal applications [43].

### 4.2. Optimization of Drug Formulation and Delivery

#### 4.2.1. Drug Properties Enhancement

Trimethyl chitosan and dextran sulfate nanocomplexes were developed through ionic interactions under varying pH conditions. These nanocomplexes demonstrated improved solubility of trimethyl chitosan compared to regular chitosan, enhancing their pharmaceutical and biomedical applications. Their enhanced solubility and mucoadhesive properties make them suitable for nasal drug delivery. These nanoparticles formed stable polyelectrolyte complexes at alkaline pH, presenting them as a reliable formulation for nasal pharmaceutical delivery [111]. Enriched chitosan nanoparticles for siRNA Delivery were specifically tailored for delivering small interfering RNA (siRNA) through polyelectrolyte complexation. Extensively evaluated biologically, these preparations underscore their potential for intranasal gene therapy applications. The enriched nanoparticles are optimized to ensure higher doses can be delivered within volumes tolerable for brain delivery, marking their importance in advancing non-invasive gene therapy delivery methods [112]. L-Cysteine modified chitosan nanoparticles for galantamine delivery were produced by conjugating thiolized chitosan with L-cysteine to enhance solubility and drug loading. Prepared via a double emulsification process, these nanoparticles exhibited prolonged drug release over 12 days, make them promising for the intranasal delivery of galantamine [113]. Xylometazoline-loaded chitosan nanoparticles were formulated to address nasal congestion, using calcium chloride cross-linking and optimized through ionotropic gelation. These nanoparticles demonstrated significant drug release and permeation through the nasal mucosa over an 8 h period, supporting their potential for effective mucosal retention and congestion treatment [44]. Chitosan-coated oil nano capsules for nasal peptide delivery were developed using the solvent displacement technique to enhance the nasal delivery of salmon calcitonin. These CS-coated oil nano capsules significantly enhanced and prolonged the hypocalcemia effect compared to uncoated nanodroplets or solutions. The chitosan coating effectively enhanced nasal absorption of salmon calcitonin, leading to more effective and prolonged hypocalcemia effects, indicating the potential of these nano capsules for nasal peptide drug delivery [114].

#### 4.2.2. Delivery Systems Optimization: Stability and Compatibility of Nanoparticles

Crosslinked chitosan nanoparticles, prepared using 2,6-pyridinedicarboxylic acid for crosslinking, demonstrated stability across various temperatures. The consistent size and charge, along with good cytocompatibility, indicate their potential utility as a drug carrier for intranasal delivery [115].

Table 4 presents various applications of chitosan nanoparticles in drug delivery, highlighting their effectiveness in improving the delivery and bioavailability of therapeutic agents. From Alzheimer’s medication to antiretroviral drugs, these nanoparticles demonstrate enhanced systemic absorption, sustained release, and targeted delivery for a range of pharmaceuticals. The table includes details such as the drug delivered, and potential therapeutic benefits, supported by corresponding references.

## 5. Specific Treatment and Conditions

### 5.1. Disease-Specific Treatments

#### 5.1.1. Viral Infections including COVID-19

**Figure 5:**
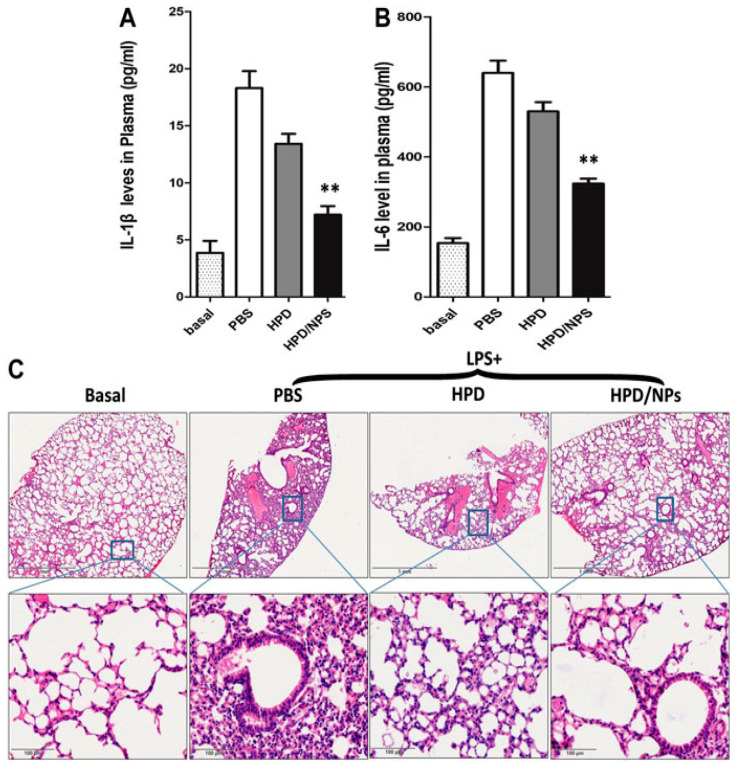
Impact of HPD and HPD/NPs on LPS-induced inflammation in mice. At 3 h post-LPS, PBS (vehicle), HPD, or HPD/NPs were nasally administered to mice. Lung tissues were collected at 24 h post-LPS challenge. (**A**) Expression levels of IL-1β and (**B**) IL-6 in mouse plasma at 24 h post-LPS challenge (n = 4/group; ** *p* < 0.001 vs. PBS vehicle and vs. HPD). (**C**) Representative micrographs of H&E-stained lung tissue cross-sections at 24 h post-LPS challenge. Scale bar, 1 mm (**upper row**) or 100 μm (**lower row**) [117].

Favipiravir-loaded chitosan–alginate nanoparticles were expertly engineered using response surface methodology to enhance the transmucosal delivery of Favipiravir (FVR). These mucoadhesive nanoparticles, termed MCS-ALG-NPs, significantly enhanced the antiviral effects of Favipiravir compared to its free form. The optimized nanoparticles proved particularly effective in increasing favipiravir’s ability to inhibit viral replication, showcasing their enhanced activity against COVID-19 and potential as a therapeutic tool in managing coronavirus infections [35]. SARS-CoV-2 spike receptor-binding domain protein was encapsulated in mannose-conjugated chitosan nanoparticles, coupled with the Toll-like receptor 9 agonist, CpG55.2, as an adjuvant. Intranasal administration of this vaccine formulation in BALB/c mice provided significant protection against SARS-CoV-2 infection in Syrian hamsters. Although the intranasal vaccination with these nanoparticles showed protective effects against SARS-CoV-2, it did not prevent viral transmission to naive animals. This indicates that while the vaccine formulation is effective in protecting against the disease, further optimization is needed to prevent transmission [37]. Hesperidin–chitosan nanoparticles for acute lung injury were developed to enhance the nasal delivery of hesperidin, with the goal of increasing cellular uptake and maximizing anti-inflammatory effects in the lungs. In a mouse model of lung inflammation, these nanoparticles significantly inhibited lung injury more effectively than free hesperidin, demonstrating their potential as a treatment for acute lung injury (ALI) and cytokine storm syndrome, conditions also pertinent to COVID-19 [117]. Figure 5 illustrates the in vivo inhibition of lipopolysaccharide-induced cytokine storm by these nanoparticles.

#### 5.1.2. Respiratory Diseases

Chitosan–DNA nanospheres targeting respiratory syncytial virus (RSV) were used in intranasal gene transfer experiments, delivering plasmid DNAs encoding all RSV antigens except for the L antigen. This approach led to significant reductions in viral titers and antigen loads in mice, while also inducing RSV-specific antibodies and T-cell responses. A single administration resulted in a notable decrease in viral titers and load, accompanied by a strong immune response, demonstrating their potential effectiveness [36]. A chitosan nanoparticle-adjuvanted chlamydia vaccine was developed, employing a novel immunization strategy that involves both intramuscular and intranasal administration of Chlamydia psittaci antigens. This dual-route approach significantly enhanced both humoral and cellular immune responses and effectively protected against respiratory infections by markedly reducing bacterial load and inflammation in the lungs. The simultaneous intramuscular and intranasal (SIM) immunization with these CNP-adjuvanted antigens induced higher levels of IgG and secretory IgA, and decreased bacterial load and inflammation in the lungs, underscoring its elevated protective responses against Chlamydia psittaci infection [118]. Self-assembled chitosan nanoparticles were employed for the intranasal delivery of recombinant interleukin-17 receptor C (IL-17RC) in a murine model of asthma. These nanoparticles effectively delivered IL-17RC, leading to a significant reduction in mucus secretion, airway inflammation, and cytokine levels. This strategy showed potential as a new method for asthma management, as it notably reduced mucus secretion and inflammatory cell infiltration in the airways, thus alleviating key inflammatory cytokines and symptoms in the murine asthma model [45]. Foot and mouth disease virus was encapsulated in fungal chitosan nanoparticles, which were prepared using fungal chitosan extracted from biomass and processed via ionic gelation. Utilizing fungal chitosan as a mucosal immunoadjuvant for intranasal vaccination induced higher serum and mucosal IgA levels in guinea pigs. The fungal chitosan nanoparticles stimulated both humoral and mucosal immunity following intranasal administration, showing efficacy comparable to that of intraperitoneally administered fluid vaccines. This highlights the potential of fungal chitosan as an effective mucosal immunoadjuvant [119].

#### 5.1.3. Psychiatric Disorders

Lurasidone hydrochloride was encapsulated in chitosan nanoparticles using ionic gelation, a process optimized through a Box–Behnken design. This formulation significantly enhanced nasal permeation, suggesting it could improve the delivery of lurasidone to the brain, potentially offering an effective treatment for psychiatric conditions such as schizophrenia [24]. Chitosan-grafted PLGA nanoparticles were developed for intranasal delivery using carbodiimide-mediated amide bond formation to enhance mucoadhesive properties. These nanoparticles, containing chlorpromazine hydrochloride, showed improved mucoadhesion, satisfactory ex vivo permeation, and maintained robust stability during testing. This formulation could serve as an effective intranasal drug delivery system for psychiatric medications [120]. Starch nanoparticle/O-carboxymethyl chitosan hydrogels were developed for intranasal delivery of the antipsychotic peptide PAOPA, a positive allosteric modulator of the dopamine D2 receptor. This system formed in situ-gelling and degradable hydrogels, showing high nasal mucosal retention and controlled release of PAOPA, potentially reducing the frequency of administration and drug doses required in schizophrenia treatment. This hydrogel system alleviated behavioral abnormalities in a schizophrenia model for up to 72 h [121]. Risperidone-loaded chitosan nanoparticles were created for intranasal delivery aimed at treating CNS disorders. Produced using ionic gelation of chitosan with tripolyphosphate and stabilized with Tween 80/Poloxamer 188, this formulation allowed for controlled drug release and enhanced delivery to brain tissues. Nasal administration of these nanoparticles showed improved brain delivery and efficacy in treating psychosis in an animal model, suggesting their potential for managing conditions such as schizophrenia [25]. Chitosan–insulin transfersomes (Transfersulin) were formulated as nanovesicles using Tween 80 and chitosan to enhance their elasticity and facilitate the nasal delivery of insulin for Alzheimer’s treatment. Produced through film hydration and characterized by TEM and FTIR, these nanoparticles exhibited effective brain delivery via nasal uptake, showing enhanced brain uptake, sustained retention, and neuroprotective effects [122]. Estradiol-loaded chitosan nanoparticles were prepared through ionic gelation to improve nasal absorption and brain targeting. Intranasal administration of estradiol-loaded CS-NPs resulted in significantly higher CSF concentrations compared to intravenous administration, indicating effective brain targeting via nasal delivery and highlighting their potential in CNS disorder management [123]. Rosmarinic acid was incorporated into chitosan-coated nano emulsions for nasal delivery, designed to provide protective effects against neuroinflammation and oxidative stress in an LPS-induced rat model. The nasal administration of these rosmarinic acid-loaded chitosan-coated nano emulsions (RA CNE) showed neuroprotective effects, improving memory, reducing neuroinflammation, and mitigating oxidative stress in rats, highlighting their therapeutic potential [124].

#### 5.1.4. Emergency and Acute Treatments

Midazolam-loaded chitosan nanoparticles were investigated for their capacity to enhance brain transport via the intranasal route. Formulated by ionic gelation, these nanoparticles displayed superior brain delivery compared to intravenous administration. This indicates their potential for treating seizure emergencies by exploiting the direct nose-to-brain pathway. Midazolam-loaded chitosan nanoparticles demonstrated enhanced brain delivery and higher bioavailability compared to intravenous and other intranasal solutions, suggesting their ability to improve therapeutic outcomes for emergency seizure treatments [125].

#### 5.1.5. Neurodegenerative Diseases

p38 MAPK Inhibitor-loaded chitosan nano capsules, encapsulating PH797804 using nano emulsion technology, underwent comprehensive in vitro, ex vivo, and in vivo studies in a mouse model of Alzheimer’s disease. The intranasal administration of these nano capsules effectively reduced p38 MAPK activity in the brain, suggesting a potential method to mitigate the systemic side effects commonly associated with this class of drugs. The encapsulated inhibitor retained its activity and demonstrated effective tissue penetration, indicating a promising strategy for targeted brain delivery to alleviate symptoms associated with neurodegenerative diseases [126]. siRNA-loaded chitosan nanoparticles for Huntington’s disease were developed to evaluate the kinetics of HTT mRNA knock-down in YAC 128 mouse brains following intranasal dosing. Employing mathematical modeling, these nanoparticles helped establish dosing schedules that effectively lower therapeutic gene expression. This approach elucidated the kinetics and cumulative effects of multiple intranasal administrations, aiding in the prediction and optimization of dosing schedules for therapeutic gene suppression [127]. siRNA-loaded chitosan nanoparticles targeting galectin-1 for glioblastoma were designed to deliver siRNA targeting galectin-1 intranasally, achieving rapid delivery to the central nervous system and significant reduction in Gal-1 expression in tumor cells. These results demonstrate the potential of these nanoparticles as an effective treatment for glioblastoma multiforme with minimal systemic effects. The successful delivery and significant reduction in Gal-1 expression highlight the potential for treating glioblastoma via non-invasive intranasal delivery [128]. Rutin-loaded chitosan nanoparticles (RT-CS-NPs) were prepared using the ionotropic gelation method. The quantification of rutin in brain homogenates was conducted using UHPLC/ESI-QTOF-MS/MS, indicating effective brain delivery of rutin following intranasal administration. The optimal extraction method utilized a mixture of ethyl acetate and acetonitrile, achieving excellent recovery and a linear dynamic range for rutin concentration determination in the brain. This method ensures that RT-CS-NPs efficiently deliver rutin to the brain, demonstrating their potential for enhancing drug delivery via the intranasal route [129].

#### 5.1.6. Pain Management

Tapentadol hydrochloride-loaded chitosan nanoparticles were formulated using ionotropic gelation. Post-intranasal delivery, these nanoparticles achieved high brain concentrations compared to other administration routes, underscoring their effectiveness in managing pain. The enhanced brain concentrations and prolonged analgesic activity compared to a drug solution highlight their potential for effective pain management through nose-to-brain delivery [27]. Cyclobenzaprine hydrochloride-loaded thiolated chitosan nanoparticles exhibited significant drug permeation and reduced toxicity on nasal epithelial cells. Administered intranasally in N-Methyl-D-Aspartate (NMDA)-induced models, these nanoparticles enhanced brain uptake and reduced hyperalgesia, confirming their enhanced trans-mucosal permeability and targeted delivery capabilities for hydrophilic drugs. This improvement in brain uptake not only boosts the drug’s effectiveness but also provides prolonged pain relief, presenting a promising approach for intranasal pain management [26].

Table 5 provides an overview of chitosan nanoparticles’ applications in treating respiratory and central nervous system disorders. From antiviral effects against COVID-19 to targeted drug delivery for neurological disorders, these nanoparticles showcase promising therapeutic interventions. The table highlights the drug delivered, along with specific therapeutic effects and potential applications, supported by corresponding references.

## 6. Testing

To effectively evaluate nano platforms, a comprehensive suite of tests is necessary, encompassing both characterization and application-specific assessments to ensure functionality and safety.

The initial phase of testing should focus on the detailed characterization of nanoparticles. This includes determining particle size, surface charge, and morphology, critical for understanding the basic properties that affect behavior in biological systems [24,25,27,41,42,43,44,111,115,116,119,122,123,125,129]. Advanced microscopy and spectroscopy techniques such as Scanning Electron Microscopy (SEM), Dynamic Light Scattering (DLS), and Fourier Transform Infrared Spectroscopy (FTIR) should also be employed to provide deeper insights into the physical and chemical properties of the nanoparticles [8,20,25,28,44,109].

In vitro evaluations are essential to determine the stability, release profiles, and cytotoxicity of nanoparticles. These tests assess how nanoparticles behave under different conditions and their potential toxicity to cells, which is crucial for predicting in vivo interactions [22,24,25,41,42,46,113,115,119,122,126].

In vivo studies should then be conducted to evaluate pharmacokinetics, bioavailability, and therapeutic efficacy. These studies are pivotal in understanding how nanoparticles distribute within the body, their site-specific drug delivery capabilities, and overall effectiveness in treatment modalities [22,25,27,113,116,117,118,122,124,125,126]. Behavioral and histological studies also play a role in assessing the functional impacts of nanoparticle treatments, particularly in disease models [25,27,58,110,120,121,122].

Immunogenicity and vaccine development are other crucial areas of testing, requiring the evaluation of immune responses to nanoparticle-based vaccines and comparisons across different administration routes. These studies help determine the efficacy of nanoparticles as vaccine carriers and their ability to elicit desired immune responses [13,29,37,86,89,118,119].

Furthermore, disease-specific testing is vital for targeted therapeutic applications, such as in models of Alzheimer’s disease, asthma, schizophrenia, and other conditions. These tests help establish the efficacy of nanoparticles in treating specific diseases and understanding their interactions within disease-specific contexts [36,45,117,118,121,124,126,127,128].

Ex vivo and permeation studies should be included to assess mucoadhesion, transmucosal permeability, and the overall behavior of nanoparticles in interacting with biological barriers. Such studies are essential for applications involving targeted delivery, such as nasal or oral routes [22,24,25,27,44,116,120].

Lastly, the development and validation of analytical methods are necessary to accurately measure and analyze nanoparticles and their payloads. This includes optimizing extraction and quantification techniques and ensuring precise measurement capabilities throughout the development and application phases [129].

Collectively, these tests provide a thorough and comprehensive evaluation of nanoparticle platforms, facilitating advancements in their development and application in various therapeutic and diagnostic fields.

## 7. Collective Outcomes

Enhanced drug delivery to the brain using technologies like chitosan-coated nanoparticles [10,11,61] and nano emulsions [56] significantly improves targeting and efficiency, crucial for treating CNS disorders such as Alzheimer’s disease and glioblastoma. These advancements are vital for overcoming the blood–brain barrier and delivering effective treatments.

Chitosan-based nanoparticles have shown considerable promise in treating neurodegenerative diseases like Alzheimer’s [5,6,8,18] and Parkinson’s [9,10,11,20,46], offering sustained and controlled drug release that enhances treatment outcomes and patient compliance. Such targeted delivery systems are particularly beneficial for drugs with narrow therapeutic windows, reducing systemic side effects and improving safety.

In gene therapy, chitosan nanoparticles facilitate direct genetic modifications within the CNS, opening new avenues for treating genetic disorders [65,66]. Additionally, these nanoparticles have propelled the development of nasal vaccines, improving immune responses against various pathogens and enabling the customization of vaccines for diseases like influenza [13,14,17,28,29,74,77,99] and hepatitis B [30,31,32,33,34,68].

Overall, chitosan nanoparticles have enhanced the delivery and efficacy of treatments across a range of conditions, including diabetes [38,39,40,42,108], hypertension [110], neurological and psychiatric disorders [22,24,25,58,122], pain management [27], and infectious diseases like RSV [36] and SARS-CoV-2 [37], significantly improving therapeutic outcomes.

## 8. Limitations and Future Directions

Scaling chitosan-based nanoparticles from laboratory to industrial production presents notable challenges, including maintaining consistent nanoparticle size, drug loading, and release characteristics. Precise controls are essential to ensure product uniformity, stability, and bioactivity, making large-scale production complex. Furthermore, the regulatory frameworks for chitosan-based nanoparticles are still evolving, potentially delaying their clinical applications. Concerns about long-term safety and potential toxicity, particularly with repeated administration, necessitate thorough investigations to confirm their safety. Most chitosan nanoparticle research is currently in the preclinical stage [5,10,21,35,62,63,99,121], highlighting significant gaps in transitioning to human trials. Clinical validation of their effectiveness and safety is crucial and involves extensive, costly testing. The production of chitosan nanoparticles entails complex processes that require optimization for consistent quality and efficient drug loading, which is critical for practical clinical use.

Extensive clinical trials are necessary to validate the efficacy and safety of nanomedicines in humans. These trials are crucial for translating preclinical findings into clinical successes. Ongoing collaboration across fields such as pharmacology, nanotechnology, and biomedical engineering is essential to address the challenges of nanoparticle design and drug delivery. Innovation in developing new materials for nanoparticles that improve biocompatibility, targeting accuracy, and therapeutic efficacy is crucial. Focusing on creating environmentally responsive materials is also recommended. Clear regulatory guidelines tailored to nanomedicines will help accelerate their development and market access. Ensuring safety, especially with long-term nanoparticle exposure, is paramount. Broadening research to encompass more diseases and personalizing medicine using chitosan nanoparticles can significantly enhance treatment outcomes, making this approach vital for effectively targeting complex diseases.

## 9. Conclusions

The advancements in chitosan-based nanoparticles have shown significant promise in enhancing drug delivery across the blood–brain barrier, targeting neurodegenerative diseases, and improving vaccine efficacy. However, challenges such as scalability, regulatory uncertainties, and limited clinical trials highlight the need for further development. Future directions should focus on advancing clinical trials to validate efficacy and safety, fostering interdisciplinary collaboration to overcome technical and manufacturing hurdles, and innovating in nanoparticle design and regulatory frameworks. Addressing these issues will be crucial for transitioning from preclinical successes to robust clinical applications, ultimately making these technologies viable for widespread therapeutic use.

## Figures and Tables

**Table 1:** Diverse applications of chitosan-based nanoparticles in brain targeting and drug delivery.

Chitosan Nano System with Active Loaded	Application and Properties	Ref.
Chitosan-decorated PLGA nanoparticles with curcumin (Cur)	General encapsulation efficiency and particle size characterization; size: 200 nm, entrapment efficiency: 75%	[5]
Lactoferrin-conjugated N-trimethylated chitosan surface-modified PLGA with huperzine A (HupA)	Enhanced mucoadhesion and targeting; size: 153.2 nm, entrapment efficiency: 73.8%, zeta potential: +35.6 mV	[6]
Thiolated chitosan nanoparticles with selegiline hydrochloride	Nasal delivery for depression treatment; size: 215 nm, entrapment efficiency: 70%, zeta potential: +17.06 mV	[7]
GH-loaded chitosan and chitosan–alginate particles with galantamine hydrobromide (GH)	Drug delivery with enhanced stability; size: 240 nm and 286 nm	[8]
Lecithin–chitosan hybrid nanoparticles with piribedil	Increased brain bioavailability, sustained release; particle size: 147 nm, drug loading capacity: 12%	[9]
Chitosan-coated nanostructured lipid carriers with glial cell-derived neurotrophic factor (GDNF)	Neuroprotection and behavioral improvement in neurological disorders; particle size: 130 nm, high encapsulation efficiency	[10]
Chitosan-coated PLGA nanoparticles with ropinirole hydrochloride	Rapid drug release for efficient drug delivery; size: 468.0 nm, zeta potential: +54.4 mV	[11]
Chitosan nanoparticles loading cyclovirobuxine D	Sustained release and increased brain targeting; diameter: 235.37 ± 12.71 nm, entrapment efficiency: 62.82%	[12]
Carboxymethyl chitosan nanoparticles with carbamazepine	Enhanced brain delivery via intranasal route for epilepsy treatment; size: 218.76 ± 2.41 nm, drug loading: about 35%, entrapment efficiency: approximately 80%	[15]
Intranasal chitosan nanoparticles with piperine	Brain-targeting via intranasal delivery; size: 248.50 nm, entrapment efficiency: 81.70%, zeta potential: +56.30 mV	[18]
Chitosan nanoparticles for sitagliptin delivery	Enhanced nasal absorption and brain targeting for Alzheimer’s disease treatment; size: 188.4 ± 48.1 nm, zeta potential: 20.8 mV	[19]
Chitosan nanoparticles with bromocriptine (BRC)	Enhanced nasal permeability and drug delivery; particle size: approx. 161.3 nm, encapsulation efficiency: 84.26%	[20]
Chitosan-lecithin nanoparticles loaded with phenytoin (PHT)	Sustained release and increased brain levels intranasally; average dynamic size: approx. 600 nm, encapsulation efficiency: over 60%	[21]
Chitosan-coated intra nasal Ropinirole nano emulsion	Potential for Parkinson’s management via enhanced brain delivery; particle size around 59 nm	[22]
Chitosan-g-HPβCD nanoparticles with efavirenz	Enhanced entrapment and drug loading via ionic gelation; diameter: 198 ± 4.4 nm, drug loading capacity: 23.28%, entrapment efficiency: 38%	[47]
Polylactic acid nanoparticles modified with chitosan-containing neurotoxin (NT)	Enhanced brain targeting and pharmacokinetics; size: 140.5 ± 5.4 nm, zeta potential: +33.71 ± 3.24 mV, entrapment efficiency: 83.51%	[49]
Nanostructured lipid carriers overlaid with chitosan with berberine	Enhanced brain delivery and pharmacokinetics; size: 180.9 ± 4.3 nm, sustained-release, surface charge: 36.8 mV	[50]
Chitosan-coated nanostructured lipid carriers with protein	Effective brain delivery after intranasal administration; size: 114 nm, surface charge: +28 mV	[52]
Thiolated okra gum and chitosan nanoparticles	Promising brain targeting properties; size: 294.3 ± 0.3 nm, entrapment efficiency: 43.57%, zeta potential: 23.29 mV	[53]
Quetiapine fumarate-loaded chitosan nanoparticles (QF-NP) with quetiapine fumarate	Increased brain/blood ratio and nasal bioavailability; size: 131.08 ± 7.45 nm, polydispersity index: 0.252, entrapment efficiency: 89.93%	[54]
Temozolomide-loaded nano lipid chitosan hydrogel	Enhanced nasal absorption and compatibility with cell lines; size: 134 nm, polydispersity index: 0.177, encapsulation efficiency: 88.45%, drug loading: 9.12%	[55]
Mucoadhesive chitosan-coated nano emulsions with rosmarinic Acid	Nasal delivery with prolonged drug release and enhanced penetration; droplet size: 270.23 to 448.40 nm, zeta potential: 41.97 to 48.63 mV	[56]
HP-β-CD/chitosan nanoparticles with scutellarin	Enhanced brain targeting for treatment of cerebral ischemia; increased brain accumulation	[57]
Simvastatin-loaded poly-epsilon-caprolactone nano capsules coated with chitosan	Increased nasal permeation and mucoadhesive properties; size: below 220 nm, high encapsulation efficiency, controlled drug release	[59]
Bioengineered PLGA–chitosan nanoparticles with thyrotropin-releasing hormone analogues (NP-355, NP-647)	Brain-targeted antiepileptic drug delivery; sustained release capabilities	[60]
Chitosan-coated PLGA nanoparticles with carmustine	Enhanced brain permeability and delivery via nasal route; size: 208 to 421 nm, polydispersity index (PDI): 0.221 to 0.561, entrapment efficiency of 58.76% and loading capacity of 11.70%.	[61]
Curcumin-laden dual-targeting fucoidan/chitosan nanocarriers	Targeted brain delivery for inflammatory lesions, responsive properties; average particle size around 170 nm with zeta potential about 25 mV	[62]
Chitosan-coated buspirone-loaded NLCs (BPE-CH-NLCs)	High brain drug targeting efficiency via intranasal delivery; size: 190.98 ± 4.72 nm, zeta potential: +17.47 mV, entrapment efficiency: 80.53%	[63]
Thiolated chitosan–Centella asiatica nanocomposite	Brain targeting via nasal route, potential blood–brain barrier (BBB) receptor targeting; good nasal permeability, low cytotoxicity, molecular docking studies. Size: 477.1 nm and 210.5 nm. Zeta potential: 36.3 mV (blank) and −14.5 mV (CTC conjugate)	[64]
Chitosan–siRNA nanoparticle formulation with siRNA	Non-invasive brain delivery for neurodegenerative disease treatment; efficient and safe brain drug delivery, successful brain targeting and gene silencing	[65]
Chitosan–mangafodipir nanoparticles with siRNA and DNA	Non-invasive gene therapy for neurodegenerative diseases via intranasal delivery; effective in reducing GFP mRNA levels, enables the expression of RFP, visualized by 7T MRI	[66]

**Table 2:** Chitosan-based nanoparticles for nasal vaccination against infectious diseases.

Chitosan Nano System with Active Loaded	Application and Properties	Ref.
N-trimethyl chitosan nanoparticles with Influenza A subunit H3N2 antigen	Nasal vaccination inducing high immune responses; size: 800 nm, loading efficiency: 78%, loading capacity: 13% (*w*/*w*)	[13]
N-trimethyl chitosan nanoparticles with r4M2e.HSP70c antigen	Induced protection and immune response against influenza via nasal delivery; size: 200 and 250 nm, zeta potential: +30 mV, PDI: 0.1–0.2	[14]
Dry powder form of chitosan nanospheres with influenza whole virus and adjuvants	Enhanced mucosal immunization against influenza via nasal administration; size: 581.1 ± 32.6 nm, PDI: 0.478 ± 0.04, encapsulation efficiency: 33.7 ± 5.77%	[17]
Chitosan nanospheres with influenza virus, CpG ODN, and Quillaja saponin	Controlled release and reduced cytotoxicity for nasal influenza vaccine delivery; size: 581.1 ± 32.6 nm, PDI: 0.478	[28]
N-trimethyl chitosan nanoparticles with Bordetella pertussis antigens	Systemic and mucosal immunity against pertussis via nasal delivery; size: 252.8 nm, zeta potential: +30.8 mV	[31]
Glycol chitosan nanoparticles with hepatitis B vaccine	Strong systemic and mucosal immune responses, promising mucosal vaccine delivery; particle size: approximately 200 nm, positively charged surface, high loading efficacy: over 95%	[32]
N,N,N-trimethyl chitosan nanoparticles with hepatitis B virus surface antigen (HBsAg)	Enhanced stability and adjuvanticity for prolonged immunogenic response; size between 143 ± 33 nm and 259 ± 47 nm, loading efficiency: 90–97%, release: 93% of HBsAg over 43 days	[33]
Alginate-coated chitosan nanoparticles with hepatitis B surface antigen (HBsAg), CpG ODN	Enhanced systemic and mucosal immune response via nasal delivery; size: 300 and 600 nm, zeta potential: −35 mV	[68]
Chitosan and chitosan–alginate nanoparticles with compound 48/80	Improved nasal residence and adjuvant effects of antigen; mean size: around 500 nm, positive charge	[69]
Curdlan sulfate–O-linked quaternized chitosan nanoparticles with ovalbumin	Improved immunogenicity and antigen uptake via intranasal administration; zeta potential: 11.8 mV, size: 178 nm.	[70]
Low-molecular-weight chitosan nanoparticles with tetanus toxoid (TT)	Induced long-lasting humoral and mucosal immune responses in nasal immunization; size: about 350 nm, positive charge: +40 mV, loading efficiency: 50–60%	[71]
Quaternized chitosan nanoparticles with live vaccine against Newcastle disease and infectious bronchitis	Effective mucosal immunization in chickens; particle size: 103.2 ± 1.6 nm, zeta potentials: 38.1 ± 2.7 mV	[72]
Comparative study using PLGA NP, TMC NP, and TMC-coated PLGA NP with ovalbumin	Superior nasal immunization effectiveness; PLGA/TMC-loaded nanoparticles size: 448 ± 55.9 nm, zeta potential: 24.5 ± 0.90 mV, loading efficiency: 71.6 ± 6.2%. TMC-loaded nanoparticles size: 278 ± 28.8 nm, zeta potential: 10.4 ± 0.20 mV, loading efficiency: 60.2 ± 4.1%	[73]
N-trimethylaminoethylmethacrylate chitosan nanoparticles with ovalbumin	Enhanced immune responses and transport across nasal epithelium; size: 140.5 ± 1.5 nm, zeta potential: 10.3 ± 0.2 mV	[74]
Chitosan–dextran sulphate nanoparticles with pertussis toxin (PTX), Immunoglobulin-A	Potential nasal vaccine delivery system; entrapment efficiency: over 90%	[75]
Alginate-coated chitosan and trimethyl chitosan nanoparticles with PR8 influenza virus	Superior Type 1 T helper (Th1)-type immune response for intranasal vaccine delivery; more positively charged of 14.6 and 13.9 mV, respectively.	[77]
Study on the adjuvanticity of N,N,N-trimethyl chitosan with influenza virus vaccine	Influence of acetylation on adjuvant effectiveness in nasal vaccine; zeta potential: +18 mV.	[78]

**Table 3:** Versatile applications of chitosan nanoparticles in vaccine development.

Chitosan Nano System with Active Loaded	Application and Properties	Ref.
Mannose-modified chitosan nanoparticles with bovine serum albumin (BSA)	Enhanced mucosal and systemic immune responses, potential for intranasal delivery; size: 156 nm, zeta: +33.5 mV	[16]
Plasmid DNA loaded chitosan Nanoparticles with Plasmid DNA for hepatitis B	Induced protective immunoglobulin levels via nasal delivery; size: 337 ± 27 nm, encapsulation efficiencies: 96.2 ± 1.8%, zeta potential: 13.8 ± 1.5 mV	[34]
Protein-loaded N-trimethyl chitosan nanoparticles with proteins like mouse monoclonal anti-ovalbumin IgG and fluorescent-labeled (Cy-5) goat IgG anti-mouse immunoglobulin	Preservation of protein integrity and controlled release; size: 350 nm, positive zeta-potential, loading efficiency: up to 95%, loading capacity: up to 50% (*w*/*w*)	[79]
N-trimethyl chitosan nanoparticles with ovalbumin, various immunopotentiators like LPS, CpG	Tailored immunogenicity for nasal and intradermal vaccine delivery; diameter: 300–400 nm	[80]
Ovalbumin-loaded N-trimethyl chitosan nanoparticles with CpG DNA	Enhanced Type 1 T helper (Th1) immune responses, proving effectiveness for nasal vaccine delivery; size: 380 nm, zeta: +21 Mv	[81]
Covalently stabilized trimethyl chitosan-hyaluronic acid nanoparticles with ovalbumin	Enhanced stability and immunogenicity for nasal and intradermal vaccination; size: 250–350 nm, positive zeta potential, OVA loading efficiencies up to 60%	[82]
Chitosan-DNA nanoparticles with pneumococcal surface antigen A (PsaA)	Enhanced mucosal and systemic immune responses against Streptococcus pneumoniae; size: 392 nm, zeta: 12.5 mV	[83]
Chitosan/pCETP nanoparticles with DNA plasmid encoding a B-cell epitope of cholesteryl ester transfer protein (CETP)	Intranasal immunization against atherosclerosis in rabbits; size: 340.2 ± 14.6 nm, zeta: +22.9 ± 1.3 mV	[84]
Chitosan–pullulan composite nanoparticles with bovine serum albumin (BSA)	Demonstrated potential for nasal delivery of vaccines; size: 207 to 603 nm, loading efficiency: >90%	[85]
Chitosan–pullulan composite nanoparticles with diphtheria toxoid (DT)	Enhanced nasal delivery of DT, improved immunological responses; size: 239–405 nm, surface charge: +18 and +27 mV	[86]
Functionalized chitosan-based composite nanoparticles; N-2-hydroxypropyl trimethyl ammonium chloride chitosan/N,O-carboxymethyl chitosan nanoparticles with bovine serum albumin (BSA)	Enhanced mucosal and systemic immune responses for intranasal vaccination; promotes lymphocyte proliferation and secretion of pro-inflammatory factors	[87]
Mixed cross-linked dextran microspheres and loaded trimethyl chitosan nanospheres with tetanus toxoid (TT)	Enhanced systemic and mucosal immune responses in nasal vaccine delivery; loading efficiency of TT in TMC NPs: 43.25% ± 3.56, zeta potential: 3.32 ± 0.05 mV and 2.28 ± 0.07 mV	[88]
Dendritic-cell-targeted chitosan nanoparticles with Plasmid DNA targeting dendritic cells	Targeted nasal immunization against SARS-CoV, enhanced mucosal and systemic responses; encapsulation efficiency of plasmid DNA encoding N protein (pVAXN): 97.6 ± 2.1%	[89]
Mannosylated chitosan nanoparticles with DNA vaccine for tumor immunity	Enhanced tumor immunity in a mouse model via nasal mucosal delivery; average tumor weight significantly lower in the treated groups	[90]
Chitosan nanoparticles loaded with Newcastle disease virus DNA vaccine	Superior efficacy in intranasal immunization of chickens; size: 202.3 ± 0.52 nm, zeta potential: 50.8 ± 8.21 mV, encapsulation efficiency: 90.74 ± 1.10%, loading capacity: 49.84 ± 1.20%	[91]
Chitosan nanoparticles loaded with Brucella abortus malate dehydrogenase	Inducing immune responses through intranasal administration, potential mucosal adjuvant; size: <1 μm	[92]
Chitosan-DNA nanoparticles for *Campylobacter jejuni* with DNA encoding FlaA protein	Reduced bacterial colonization, enhanced systemic and mucosal immunity in poultry; size: 80–100 nm, association efficiency: 91.9%	[93]
Chitosan nanofibrous membrane for Shigella vaccine with N-terminal region of invasion plasmid antigen D (IpaD antigen)	Protection against *S. flexneri*, significant antibody responses via nasal delivery; encapsulation efficiency of N-IpaD: 64.7 ± 14.3%	[94]
Polyglutamic acid-trimethyl chitosan peptide nano-vaccine with peptide antigen	Potent systemic and mucosal antibody responses against Group A Streptococcus; zeta-potential: +36 mV, size: 201 ± 8 nm	[95]
Chitosan-based nanoparticles for Japanese encephalitis with live attenuated Japanese encephalitis vaccine	Enhanced mucosal and systemic immunity, potential non-invasive vaccination route; loading efficiency and capacity of JE-CV in CS NPs and CM NPs: 85.27–86.04%	[96]
Alginate–chitosan nanoparticles for tuberculosis Vaccine with PPE17 protein and CpG	Enhanced efficacy of BCG vaccines, strong Type 1 T helper (Th1) and IL-17 responses via nasal and subcutaneous routes; size: about 427 nm, zeta potential: −37 Mv	[97]
Chitosan–alginate nanoparticle encapsulated bee venom for porcine reproductive and respiratory syndrome virus (PRRSV)	Enhanced T cell responses and viral clearance in pigs; size of AL-BV and CH/AL-BV: 541.5 ± 50.9 nm and 434.6 ± 22.1 nm	[98]
Chitosan nanoparticles for swine influenza Vaccine (SwIAV) with inactivated influenza vaccine	Enhanced IgG and mucosal IgA responses, cross-reactive protection in pigs; encapsulation efficiency of SwIAV antigens (KAg): 67%	[99]
Chitosan nanoparticles with P10 peptide from Paracoccidioides brasiliensis	Reduction in fungal load and immune modulation via intranasal immunization; size: 220 nm, pdi below 0.5, zeta potential: +20 mV, encapsulation efficiency around 90%	[100]
Chitosan nanoparticles with fluorescent labeling and P10 peptide	Localization in upper airway, effective fungal load reduction, induced immune responses; size range: 230–350 nm, zeta potential: +20 mV	[101]
Chitosan nanoparticles with inactivated Chlamydia psittaci elementary bodies (EBs)	Enhanced immune responses and protection against lung lesions in chickens; VCG + EB-immunized chickens showed significantly reduced lesions compared to controls	[102]

**Table 4:** Chitosan nanoparticles for enhanced drug delivery.

Chitosan Nano System with Active Loaded	Application and Properties	Ref.
Chitosan nanoparticles for olanzapine delivery w	Enhanced systemic bioavailability for psychiatric medication delivery; Size ranged from 179 to 237 nm (20% loading) and 304 to 340 nm (60% loading), Encapsulation efficiency: nearly 90%	[23]
Chitosan nanoparticles for insulin delivery with human zinc insulin	Examined nasal absorption of insulin; pharmacological response observed; Size: 751.8 ± 74.7 nm, Zeta potential: 41.2 ± 0.8 mV	[38]
Chitosan nanoparticles for nasal insulin delivery	Enhanced nasal and systemic absorption of insulin; size: 300–400 nm, positive surface charge, insulin loading: up to 55% (insulin/nanoparticles *w*/*w*)	[40]
Chitosan-N-acetyl-L-Cysteine nanoparticles with insulin	Enhanced mucosal absorption and controlled insulin release; zeta potential: + 19.5–31.7 mV, insulin loading: 1.3–42%	[41]
PEG-grafted chitosan nanoparticles for insulin with pure crystalline porcine insulin	Improved nasal absorption of insulin in rabbits; size range: 150–300 nm, charge: +16 to +30 mV, loading efficiency: 20–39%	[42]
Adaptive chitosan-based nano-vehicles with cetirizine	Responsive drug release in nasal conditions, suitable for allergic therapy; size: 120 nm, zeta potential: 4 mV	[43]
Xylometazoline-loaded chitosan nanoparticles	Effective treatment for nasal congestion with high mucosal retention; size: 172 nm, PDI: 0.27, encapsulation efficiency: 90.5%	[44]
Chitosan nanoparticles for galantamine delivery	Improved delivery and bioavailability in Alzheimer’s disease models; linearity: 0.5–300 ng mL^−1^, trueness and precision: acceptable, recoveries: 85.6–114.3%	[103]
Chitosan nanoparticles for dihydroergotamine delivery	Improved systemic absorption for acute migraine treatment via nasal delivery; size: 395 ± 59 nm, 20% loading with 95 ± 13% encapsulation efficiency, bioavailability: 82.5 ± 12.3%	[104]
Chitosan nanoparticles for didanosine Delivery	Enhanced systemic and brain targeting for AIDS-related infections; brain/plasma, olfactory bulb/plasma, CSF/plasma concentration ratios significantly higher (*p* < 0.05)	[105]
Chitosan Nanoparticles for zidovudine delivery	Improved mucoadhesion and drug flux for nasal administration; Sizes: AZT-loaded NP1 (406 nm), AZT-loaded NP2 (425 nm), Entrapment efficiency: 17.58% ± 1.48 (NP1), 11.02% ± 2.05 (NP2)	[106]
Silver Nanoparticles/Chitosan/PVA/PEG hydrogel nanocomposite with silver nitrate (AgNO_3_)	Medical applications in nasal tampons with antibacterial and blood coagulation effects; Z-average: about 96 nm	[107]
Trimethyl chitosan and PEGylated nanocomplexes for Insulin with human recombinant insulin	Enhanced nasal absorption with reduced mucosal toxicity; all insulin nanocomplexes showed a 34–47% reduction in blood glucose concentration	[108]
Chitosan lactate nanoparticles with enalaprilat	Prolonged drug permeation and effective hypertension control in rats; size: 213 nm, % drug entrapment: 30.04%, zeta potential: 45.83 mV	[109]
Chitosan nanoparticles with olmesartan medoxomil	Increased bioavailability for hypertension treatment, safe for nasal mucosa; size: 240.02–344.45 nm, %EE: 75.2–83.51%, improved bioavailability by 11.3-fold	[110]
N,N,N-trimethyl chitosan and dextran sulfate nanocomplexes with mucin	Improved drug delivery due to enhanced solubility and biocompatibility; zeta potentials of native chitosan (CHT) and synthesized (TMC): 27.7 and 18.7 mV, respectively	[111]
Enriched chitosan nanoparticles with siRNA	Potentially powerful tool for intranasal gene therapy; size: 90 to 200 nm, zeta potential: +42 to +55 mV	[112]
L-Cysteine modified chitosan Nanoparticles for galantamine delivery	Prolonged release suitable for intranasal delivery; size: ~800 to ~1 mm, zeta potential: above 40 mV	[113]
Chitosan-coated oil nano capsules for nasal peptide delivery with salmon calcitonin	Enhanced and prolonged hypocalcemia effect; size: 160–250 nm, zeta potential varied by coating, positive for chitosan-PEG nano capsules	[114]
Thiolated chitosan nanoparticles with leuprolide	Sustained release and enhanced mucosal absorption for hormonal therapy; size: 252 ± 82 nm, zeta potential: +10.9 ± 4 mV, payload: 12 ± 2.8	[116]

**Table 5:** Chitosan nanoparticles for respiratory and central nervous system disorders.

Chitosan Nano System with Active Loaded	Application and Properties	Ref.
Lurasidone hydrochloride in chitosan nanoparticles	Improved nasal permeation and potential enhanced brain delivery for antipsychotic treatment; mean particle size: 154.8 nm, encapsulation efficiency: 88.5%	[24]
Risperidone-loaded chitosan nanoparticles	Controlled drug release and enhanced brain delivery for treating CNS disorders; particle size: 137.9 nm, zeta potential: +23.4 mV, encapsulation efficiency: 65.1%	[25]
Cyclobenzaprine hydrochloride-loaded thiolated chitosan nanoparticles	Enhanced brain uptake and trans-mucosal permeability; significant high drug permeation, reduced toxicity	[26]
Tapentadol hydrochloride-loaded chitosan nanoparticles	High brain concentrations post-intranasal delivery for effective pain management; particle size: 201.2 nm, zeta potential: +49.3 mV, High drug loading and entrapment efficiency	[27]
Favipiravir-loaded chitosan–alginate nanoparticles	Enhanced transmucosal delivery and anti-viral effects; superior mucoadhesion, increased deposition in nasal mucosa, 35-fold improvement over free drug; size: 233 nm, zeta potential: −21.6 mV	[35]
SARS-CoV-2 spike RBD protein in mannose-conjugated chitosan nanoparticles	Induced robust mucosal and Th1-cell responses, protection against COVID-19; average size: 290 ± 18 nm, uptake rate of RBD protein: 66.8%	[37]
Chitosan nanoparticles for intranasal delivery of IL-17RC with Interleukin-17 receptor C (IL-17RC)	Therapeutic strategy for reducing asthma symptoms; size: 212.2 nm, zeta potential: ~12 mV, encapsulation efficiency: up to 96.08 ± 3.34%	[45]
Hesperidin/chitosan nanoparticles	Enhanced anti-inflammatory effects in lungs, potential treatment for ARDS; size: 200 nm, zeta potential: +22 mV, encapsulation rate: 81.02%	[117]
Chitosan nanoparticles with Chlamydia psittaci antigens	Enhanced immune responses and protection against respiratory infection; size: 276.1 nm, zeta potential: 13.12 mV, encapsulation efficiency: 71.7%	[118]
Foot and mouth disease virus in fungal chitosan nanoparticles	Mucosal immunoadjuvant for intranasal vaccination; size range: 221.9–281.2 nm, positive charges: +7 to +13 mV, high antigen loading capacities: 93–97%	[119]
Chitosan–insulin transfersomes (transfersulin)	Enhanced elasticity and nasal delivery targeting Alzheimer’s treatment; particle size: 86 nm, zeta potential: +36.6 mV, high mucoadhesion rate	[122]
Estradiol-loaded chitosan nanoparticles	Improved nasal absorption and direct transport into CSF for CNS disorders; mean particle size: 423.41 nm	[123]
Rosmarinic acid in chitosan-coated nano emulsion w	Neuroprotective against neuroinflammation and oxidative stress; size: 310 nm, zeta potential: >35 mV	[124]
Midazolam-loaded chitosan nanoparticles	Superior brain delivery via intranasal route for treating seizure emergencies; mean size: 269.3 nm, zeta potential: +25.4 mV	[125]
siRNA-loaded chitosan nanoparticles targeting galectin-1	Rapid CNS delivery and significant reduction in Gal-1 expression in glioblastoma; Z-average: 141 ± 5 nm, PDI: 0.27	[128]
Rutin-loaded chitosan nanoparticles (RT-CS-NPs)	Effective brain delivery quantified by advanced analytical techniques; particle size within a range of 85–100 nm, PDI: 0.206	[129]
